# Chaperna-Mediated Assembly of Ferritin-Based Middle East Respiratory Syndrome-Coronavirus Nanoparticles

**DOI:** 10.3389/fimmu.2018.01093

**Published:** 2018-05-17

**Authors:** Young-Seok Kim, Ahyun Son, Jihoon Kim, Soon Bin Kwon, Myung Hee Kim, Paul Kim, Jieun Kim, Young Ho Byun, Jemin Sung, Jinhee Lee, Ji Eun Yu, Chan Park, Yeon-Sook Kim, Nam-Hyuk Cho, Jun Chang, Baik L. Seong

**Affiliations:** ^1^Department of Biotechnology, College of Life Sciences and Biotechnology, Yonsei University, Seoul, South Korea; ^2^Vaccine Translational Research Center, Yonsei University, Seoul, South Korea; ^3^Graduate School of Pharmaceutical Sciences, Ewha Womans University, Seoul, South Korea; ^4^Life Science and Biotechnology, Underwood International College, Yonsei University, Seoul, South Korea; ^5^Division of Infectious Diseases, Department of Internal Medicine, Chungnam National University School of Medicine, Daejeon, South Korea; ^6^Department of Microbiology and Immunology, Seoul National University College of Medicine, Seoul, South Korea; ^7^Department of Biomedical Sciences, Seoul National University College of Medicine, Seoul, South Korea

**Keywords:** nanoparticle, virus-like particle, chaperone, ferritin, Middle East respiratory syndrome coronavirus, receptor-binding domain, Lysyl-tRNA synthetase, RNA-interacting domain of human LysRS

## Abstract

The folding of monomeric antigens and their subsequent assembly into higher ordered structures are crucial for robust and effective production of nanoparticle (NP) vaccines in a timely and reproducible manner. Despite significant advances in *in silico* design and structure-based assembly, most engineered NPs are refractory to soluble expression and fail to assemble as designed, presenting major challenges in the manufacturing process. The failure is due to a lack of understanding of the kinetic pathways and enabling technical platforms to ensure successful folding of the monomer antigens into regular assemblages. Capitalizing on a novel function of RNA as a molecular chaperone (chaperna: chaperone + RNA), we provide a robust protein-folding vehicle that may be implemented to NP assembly in bacterial hosts. The receptor-binding domain (RBD) of Middle East respiratory syndrome-coronavirus (MERS-CoV) was fused with the RNA-interaction domain (RID) and bacterioferritin, and expressed in *Escherichia coli* in a soluble form. Site-specific proteolytic removal of the RID prompted the assemblage of monomers into NPs, which was confirmed by electron microscopy and dynamic light scattering. The mutations that affected the RNA binding to RBD significantly increased the soluble aggregation into amorphous structures, reducing the overall yield of NPs of a defined size. This underscored the RNA-antigen interactions during NP assembly. The sera after mouse immunization effectively interfered with the binding of MERS-CoV RBD to the cellular receptor hDPP4. The results suggest that RNA-binding controls the overall kinetic network of the antigen folding pathway in favor of enhanced assemblage of NPs into highly regular and immunologically relevant conformations. The concentration of the ion Fe^2+^, salt, and fusion linker also contributed to the assembly *in vitro*, and the stability of the NPs. The kinetic “pace-keeping” role of chaperna in the super molecular assembly of antigen monomers holds promise for the development and delivery of NPs and virus-like particles as recombinant vaccines and for serological detection of viral infections.

## Introduction

Various types of viral vaccines have been developed over the last century with a wide spectrum of efficacy and safety (1, 2). The manufacturing of most conventional vaccines—live attenuated, inactivated, or subunit vaccines—invariably require the culturing of infectious viruses in cell substrates (3). Despite dedicated efforts, conventional cell culture often fails to produce sufficient amounts of virus for evaluating the immunogenicity, protective efficacy, and safety of viral vaccines. Moreover, some emerging viruses cause high-mortality rates, without options for treatment or prophylaxis, necessitating their manipulation, and manufacture under stringent bio-safety environment (4). Not surprisingly, alternative technologies that circumvent these limitations are a high priority in the areas of vaccine development and production. Nanoparticles (NPs), virus-like particles (VLPs), and assembly of multimeric peptides each provide attractive platforms for vaccine design (5).

Virus-like particles and NPs structurally resemble infectious virions, but are non-infectious due to the lack of viral genomes. Recombinant surface antigens from natural virions are assembled into highly ordered conformations as empty particles devoid of genetic material. Antigenic epitopes are presented on the multivalent and highly repetitive outer structure of the NPs, which leads to the crosslinking of B-cell receptors and the induction of long-lasting immune responses (6–8). By mimicking the morphology of the natural infectious virions, the regularly assembled particles are highly immunogenic, and are amenable to diagnostic and prophylactic exploitation. Among the simplest targets are the VLPs of non-enveloped viruses, such as hepatitis E virus or human papilloma virus, and are composed purely of viral capsid proteins (9–11).

In contrast to non-enveloped viruses, where virion assembly is exclusive to capsid proteins, enveloped viruses (e.g., coronavirus or flavivirus), require an additional membrane component for assembly into mature virions. Consequently, in enveloped VLPs, the assembly of matrix proteins provides a molecular scaffold, and viral antigens are embedded into lipid membranes. Different types of glycoproteins may be embedded in the lipid membrane as target antigens for generating immunological responses (12). However, this process requires multiple proteins (surface antigens and matrix proteins), and the enveloped VLPs are not structurally uniform and are difficult to characterize. A promising alternative is to present the target antigens on the surfaces of self-assembled NPs, which, in lieu of lipid membranes, serve as the macromolecular scaffold for the presentation of the antigens of interest.

In developing NP vaccines, consideration should be given regarding the selection of a robust and faithful system for NP assembly that enables the cost-effective development and delivery of vaccines in a timely manner. Structure-based approaches *in silico* and their underlying principles are relatively advanced for NP assembly (13–15). Most of the approaches consider the thermodynamic stability of the final assembled NPs, without due recognition for the kinetic complexities controlling regular assemblage over random interactions that lead to misfolded aggregations. Therefore, it is not surprising that most engineered NPs are refractory to soluble expression, which presents practical challenges in production, both at a laboratory-scale and in commercial manufacturing processes. This problem becomes augmented when expressed in bacterial hosts because of a lack of folding assistance in the bacterial cytoplasm for viral antigens. Therefore, due to advantages in assisted folding, post-translational modifications, and the possibility of generating multiple-component NPs and VLPs, eukaryotic hosts such as yeast, insects, and mammalian cells have been favored over bacterial hosts (16–18). However, these systems are significantly more expensive than bacterial systems, are more time-consuming, and the down-stream processes are usually more complex. Moreover, the purification of VLPs from insect cell systems poses a challenge due to similar physicochemical properties between the VLPs and the baculoviruses (1, 16). Bacterial systems, if available, would provide a cost-effective means to develop and deliver vaccines, as well as sero-diagnostic antigen kits used to diagnose-specific infection diseases.

Middle East respiratory syndrome (MERS) was first reported in Saudi Arabia in 2012 and has caused multiple cases of infection with high mortality in Europe and Asia (19, 20). MERS is caused by MERS-coronavirus (MERS-CoV), which can be transmitted from camels to humans, and from humans to other humans (21, 22). Worldwide transmission is increasing in direct household and community-wide transmission, as well as in nosocomial settings, as exemplified in a 2015 outbreak in Korea (23, 24). Neither effective vaccines nor therapeutic interventions are currently available. Because of this, assembly of MERS-CoV antigens into immunologically relevant conformation as NPs would be of interest and may be helpful in developing vaccines, sero-diagnostic tools, and therapeutic monoclonal antibodies.

In the current study, we present a novel bacterial NP of MERS-CoV antigen using ferritin as a molecular scaffold for self-assembly. Ferritin, which is present in most living organisms, has 24 identical subunits that spontaneously self-assemble and form NP complexes with internal and external diameters of 8 and 12 nm, respectively (25, 26). Previous studies show that ferritins of *Helicobacter pylori* from a human isolate can be used as scaffold for HIV and influenza NP vaccines, using eukaryotic host cells such as human embryonic kidney cells (HEK293F or HEK293S) (27, 28). Likewise, bacterioferritin (FR), which self-assembles into nanocages with octahedral symmetry, has also been evaluated as a potential drug delivery system (29). However, viral antigens of human pathogens are prone to misfolding into aggregates, which necessitates chemical refolding of the insoluble aggregates in order to regain solubility and to allow regular assembly of the antigen (30, 31). In addition, displaying antigens on the surface of multi-molecularly assembled scaffolds in bacterial hosts remains a daunting challenge.

We hypothesized that NPs displaying the receptor-binding domain (RBD) of the spike protein from MERS-CoV could be produced in a bacterial system by harnessing the function of a molecular chaperone. Conventionally, protein folding and the prevention of non-functional aggregation have been ascribed to molecular chaperones (32–34). Recently, it has been shown that RNA molecules are able to provide novel functions as molecular chaperones (35–37). Based on novel findings, the concept of chaperna (chaperone + RNA) function was established (38). In this report, chaperna function was harnessed for the folding and assembly of hybrid ferritin monomers into NPs using a bacterial expression system. We also demonstrated that the biophysical properties, including solubility, yield, and stability of MERS-CoV NPs, could be improved by properly controlling the RNA-binding affinity, and the concentrations of Fe^2+^ and salts. The chaperna-based NP assembly may prove to be a versatile tool for developing and delivering recombinant vaccines and for serological detection of emerging/re-emerging viruses.

## Materials and Methods

### Ethics Statement

All animal research was performed according to the guidelines of Ministry of Food and Drug Safety of Republic of Korea. All experiments were approved by the YLARC Institutional Animal Care and Use Committee (IACUC; permit number: IACUC-A-201710-377-01). Six-week-old female BALB/c mice were purchased from ORIENT BIO Inc. (Seoul, Korea). Sera from the recovered MERS patients were used after ethical approval granted by the institutional review boards of Chungnam National University Hospital (IRB no. 2015-08-029) and Seoul National University Hospital (IRB no. 1509-103-705). This study was performed in accordance with the ethical standards laid down in the 1964 declaration of Helsinki and all subsequent revisions. Informed consent was obtained from all patients participated in this study.

### Construction of Expression Vectors

**Figure 1:**
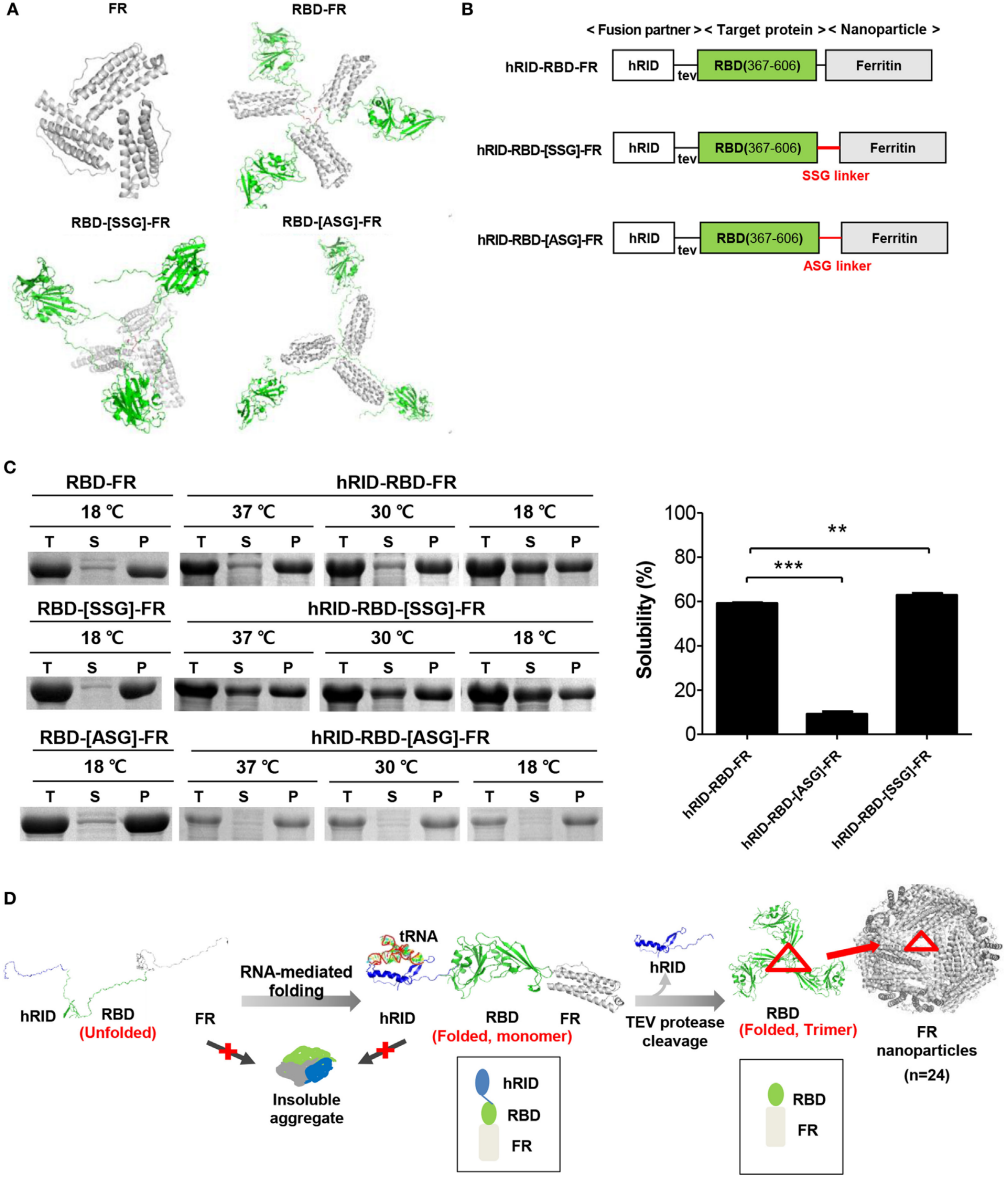
Design and soluble expression of Middle East respiratory syndrome-coronavirus (MERS-CoV) receptor-binding domain (RBD) nanoparticles (NPs) using the chaperna-based expression platform. **(A)** Computational modeling of FR, RBD-FR, RBD-[SSG]-FR, and RBD-[ASG]-FR. The hRID domain, RBD, FR, and linkers are colored as blue, green, gray, and red, respectively. **(B)** Schematic diagram of the expression vector system. The two linkers are shown in red, and tev indicates the TEV protease recognition site. **(C)** Expression of hRID-RBD-FR in the presence or absence of the hRID fusion partner and linkers. The proteins were expressed at various temperatures (37, 30, and 18°C) and the cell lysates were separated into total (T), soluble (S), and insoluble (P) fractions by centrifugation (left panel). The solubility of each protein expressed at 18°C was measured by a gel densitometer and the data were summarized and shown in the right panel (*n* = 3). Statistical significance (**p < 0.01, ***p < 0.001) was indicated for the samples compared with the control using a two-tailed Student’s *t*-test. **(D)** Illustration of MERS-CoV RBD-FR NPs using the chaperna-based hRID fusion partner. The hRID facilitated folding of the aggregation-prone RBD-FR through interaction with RNA. The monomer of RBD-FR formed a properly folded trimeric structure by cleaving hRID with TEV protease. Eight trimers assembled and formed into MERS-CoV-like NPs. Red triangles indicate the RBD trimer on the FR NPs.

The expression vector pGE-hRID(3) was constructed from the parental vector pGE-LysRS (3) (39). The pGE-LysRS(3) vector was enzymatically cut with NdeI and KpnI. The PCR product of hRID, which carries the TEV protease cleavage site and a 6-histidine tag at the C-terminus, was cut using the same restriction enzymes and the digested fragment inserted into the vector to generate pGE-hRID(3). FR (Genebank accession No. NC_000913.3) DNA was synthesized by, and purchased from, COSMO GENETECH (Korea). The DNA was cleaved with SalI and HindIII, and inserted into pGE-hRID(3) to generate hRID(3)-FR. The receptor binding domain (RBD), N-terminal residues 367–606, of the MERS-CoV S protein (GenBank accession No. AFS88936.1), was generated by gene synthesis, cut with KpnI and SalI, and inserted into hRID-FR to generate pGE-hRID(3)-RBD-FR. Linker SSG or ASG was inserted into the C-terminus of the RBD using overlapping PCR, cleaved with KpnI and SaI, and ligated into hRID-FR, generating pGE-hRID(3)-RBD-[SSG]-FR or pGE-hRID(3)-RBD-[ASG]-FR, respectively. The schematic diagrams of each expression vector are illustrated in Figure 1B. The genes of mutant hRID(2 m) (K19A and K23A) and hRID(9 m) (K19A, K23A, R24A, K27A, K30A, K31A, K35A, K38A, and K40A) were generated by gene synthesis, cleaved with NdeI and KpnI, and inserted into pGE-hRID(3)-RBD-FR, generating pGE-hRID(2 m)-RBD-FR and pGE-hRID(9 m)-RBD-FR, respectively. The mutation sites and amino acid sequences of the mutants are shown in Table S1 in Supplementary Material.

### Protein Expression and Purification

The resulting expression vectors were transformed into the *Escherichia coli* strain SHuffle^®^ T7. The cells were grown in 50 ml of LB medium with ampicillin (50 µg/ml) at 30°C overnight. Each type of transformant was inoculated into 500 ml of LB medium with ampicillin, grown at 30°C until an optical density (OD_600_) of 0.6–0.8 was reached. Protein expression was induced with 1 mM IPTG for 12 h. Each sample was harvested by centrifugation, lysed by sonication in lysis buffer (50 mM Tris–HCl, pH 7.5; 10% glycerol; 2 mM 2-mercaptoethanol; and 0.1% Tween-20). The soluble fraction of each lysate was purified on a Ni-affinity Histrap™ HP column by ATKA prime (GE Healthcare) and concentrated with Centriprep™ (Merck Millipore Ltd.). The purified proteins were treated with TEV protease to remove the fusion partner hRID. The assembled NPs were purified by gel filtration on 10/300 Superose™ 6 Increase columns (GE Healthcare).

### Homology Modeling and Trimer Simulation of RBD-FR

A homology model for the fusion complex of MERS-CoV RBD and FR was generated by MODELER, version 9.16 (Sali Lab of California) (13) using data from protein data base (PDB) for MERS-CoV RBD (PDB ID code 4kqz) and bacterial ferritin (PDB ID code 1bcf) as templates. The linker domains were improved using refinements in the loop domain (40). Energy-stable models of RBD-FR, RBD-[SSG]-FR, RBD-[ASG]-FR, and RBD-[D6]-FR for trimer structure formation were predicted using Multimer Docking software, ClusPro (ABC Group and Structural Bioinformatics Lab Boston University and Stony Brook University). The thermodynamic stabilities were calculated using the Cluspro formula for Coefficient Weights (*E* = 0.40Erep + −0.40Eatt + 600Eelec + 1.00EDARS) (41, 42).

### Characterization of NPs Using Transmission Electron Microscopy (TEM) and Cryo-Electron Microscopy (cryo-EM)

To examine the size and structure of the purified NPs, microscopic evaluations using TEM and cryo-EM were performed. For TEM analysis, a drop of the NPs was placed onto a formvar/carbon-coated TEM grid (SPL). The grid was negatively stained with 2% uranyl acetate, dried, and examined using a JEM-1011 electron microscope (JEOL) at an accelerating voltage of 80 kV. The particle sizes were calculated using Camera-Megaview III (Soft imaging system-Germany) for measuring the NPs in random image fields. For cryo-EM, the NPs were placed onto plasma-treated formvar/carbon 200 copper grid (EMS) and negatively stained with 2% uranyl acetate. The grid was accelerated at 200 kV with an FEI CryoTecnai F20 cryo-EM microscope made available through the Korean Institute of Science and Technology. The NPs were examined and photographed in high resolution.

### Dynamic Light Scattering (DLS)

Nanoparticle samples (3 ml) were placed into a Dispo-H cell, and analyzed using a Zeta-potential & Particle size Analyzer (ELS-2000ZS; Otsuka Electronics). The intensity distribution diameter of the NPs was measured twice at 25°C in water as a solvent with the sample accumulation time at 200 s.

### Effect of Salt and Fe^2+^ Concentrations on NP Assembly and Stability

Cultured cells (3 ml) were lysed with lysis buffer in the presence of various concentrations of NaCl (0, 50, 100, 150, 200, 225, 250, 275, and 300 mM) to evaluate the intracellular proteins. All samples were performed in triplicate. The cell lysates were separated into soluble and insoluble fractions by centrifugation, and the protein stabilities analyzed by sodium dodecyl sulfate-polyacrylamide gel electrophoresis (SDS-PAGE). Thus, the proteins from cell lysates (500 ml culture) were purified using HisPur™ Ni-NTA Resin (Thermo Fisher Scientific) in buffer A depending on NaCl concentration (0–300 mM). To evaluate the effects on Fe^2+^ on NP formation, cells were cultured in LB media with various concentrations of Fe^2+^ (0, 200, 500, and 1,000 µM). NP formation was examined by size exclusion chromatography (SEC), SDS-PAGE, TEM, and DLS at the various concentrations of NaCl or Fe^2+^.

### Analysis of Protein Stability and RNA Binding

The cells were harvested, sonicated with lysis buffer, and separated into soluble and pellet fractions by centrifugation. Target proteins in the soluble fraction were purified using HisPur™ Ni-NTA Resin (Thermo Fisher Scientific), following the manufacturer’s instruction. T (total lysate), S (soluble fraction), P (pellet fraction), W (wash fraction), and E (the elution fraction were analyzed by SDS-PAGE. Co-purification of the nucleic acids and proteins in the wash and elute were analyzed on a native agarose gel. The nucleic acids were visualized with ethidium bromide (EtBr), and the proteins with Coomassie staining.

### RNase A Treatment

Cultured cells (10 ml) were harvested using the same method described above. The cells were lysed with 500 µl of protein extraction reagent B-PER™ II (Thermo scientific) and separated into soluble and pellet fractions by centrifuged 12,000 rpm for 10 m. A 200 µl aliquot of each soluble fraction was further treated with 250 µg/ml of RNase A (iNtRON Biotechnology) and incubated at 37°C for 15 min. The nuclease treated samples were clarified by centrifugation at 12,000 rpm for 15 min and the soluble supernatants and the pelleted precipitates were analyzed on an SDS-PAGE gel followed by western blot analysis.

### hDPP4-Binding Enzyme-Linked Immunosorbent Assay (ELISA)

To confirm the proper folding of RBD-FR and its variant (RBD-[SSG]-FR), the binding of the purified proteins with the MERS-Cov receptor hddp4 was performed by ELISA. FR only and phosphate-buffered saline (PBS) were used as negative controls. Nunc 96-well microtiter immunoplates (Thermo Fisher Scientific) were coated with 100 ng/well of hDPP4 proteins (Abcam) and incubated at 4°C overnight. The plates were washed and blocked with 150 µl/well of blocking buffer (1% BSA) for 1 h at room temperature. RBD (SSG linker, WT, 2 m, or 9 m)-FR (100 ng/well) were added for 2 h at 37°C. An anti-penta His antibody (100 µl/well; Qiagen) was serially diluted (1/100 to 1/12,800) in TBST [50 mM Tris–Cl (pH 7.4), 0.05% Tween-20], added to the wells, and incubated for 1 h at 37°C. A secondary goat anti-mouse IgG antibody conjugated with HRP in a 100-µl volume (1:5,000, Sigma-Aldrich) was added and incubated for 1 h at 37°C. The plates were washed three times with TBST at the end of each step. After washing, 100 µl/well of substrate TMB solution (BD Biosciences) were added to the well and the plates were incubated at 37°C for 30 min in the dark. 50 µl of stop solution (2 N H_2_SO_4_) was added to the well to stop the colorimetric reaction, and the absorbance at 450 nm was measured using an ELISA reader, FLUOstar OPTIMA (BMG LABTECH).

### Serological Detection of MERS-CoV Infection

Each well of a 96-well microplates (NUNC, Roskilde, Denmark) was coated with 250 ng of four proteins [RBD-[SSG]-FR, RBD-FR(WT), RBD-FR(2 m), and RBD-FR(9 m)] and incubated overnight at 4°C. MERS-CoV RBD protein (MERS-RBD-005P; eEnzyme) was used as a positive control. Following each subsequent step, the wells were washed three times with buffer (0.05% TWEEN-20-PBS; Sigma-Aldrich, St. Louis, MO, USA). The coating antigens were removed, and the wells were blocked with PBST (5% skim milk in PBS and Tween-20) for 1 h at 37°C. After 2 h, the blocking solution was removed. Twofold serially diluted sera from four patients (CNNH-0709, 0809, 1009, and 1309) were added to each well and incubated at 37°C for 2 h. The antigen-coated wells were incubated with peroxidase-conjugated goat anti-human IgG antibody (KPL, SeraCare Life Sciences, Milford, MA, USA) at 37°C for 1 h. The primary antibody was removed and 3,3′,5,5′-tetramethylbenzidine (TMB; Sigma-Aldrich) was added to each well as colorimetric substrate. Immediately after treatment of the reactions with stopping solution (Sigma-Aldrich), the OD was read at 450 nm.

### Mouse Immunization and Sera Collection

Six-week-old female BALB/c mice were immunized with 20 µg/mouse of the RBD-FR, RBD-[SSG]-FR, or RBD protein generated as described above, or with commercially available MERS-CoV RBD protein (MERS-RBD-005P; eEnzyme) as antigen in BSL-2 facility in YLARC. Antigens were diluted in PBS. For the first group, equal volume of MF59 adjuvant (AddaVax, Cat. No vac-adx-10) (43) was mixed by pipetting. For the other group, equal volume of antigens and alum adjuvant (Thermo Fisher Scientific) were mixed by pipetting following the manufacturers’ protocol. PBS plus adjuvant and FR were used as negative controls. The immunized mice were boosted twice with intramuscular injections on days 14 and 28. Mice were anesthetized on days 27 and 41 for ocular bleeding from the orbital sinus (Figure S10 in Supplementary Material). Immune sera were processed by centrifugation of the collected blood at 12,000 × *g* for 30 min. The spleen and the BALF (bronchoalveolar lavage) were obtained at 7 days after the last immunization from sacrificed mice. BALF was taken by washing the airways with 1 ml of PBS.

### Flow Cytometric Analysis

T-cell population from immunized mice were analyzed by Flow cytometric analysis (43, 44). The spleens were taken at 7 days after the last immunization from the sacrificed mice. To obtain single-cell suspensions, the tissues were homogenized and passed through 70 µm cell strainers (SPL). After centrifugation, erythrocytes were removed by Red Blood Cell Lysing Buffer (Sigma). The cells were washed and resuspended in Iscove’s Modified Dulbecco’s Media containing 10% FBS. For intracellular cytokine staining, the splenocytes were stimulated with 10 µg/ml RBD protein or phorbol myristate acetate/ionomycin in the presence 10 ng/ml recombinant human IL-2 (BioLegend) and Brefeldin A (1:1,000; eBioscience) at 37°C for 5 h. After stimulation, the cells were blocked with rat anti-mouse CD16/CD32 (BD Biosciences) and surface stained with anti-CD8 (FITC, clone 53-6.7; BioLegend) and anti-CD4 (PE/Cy7, clone GK1.5; BioLegend) at 4°C for 30 min. The stained cells were fixed in FACS lysing solution (BD Biosciences) at room temperature for 20 min, and permeabilized with FACS buffer (0.5% FBS, 0.1% NaN3 in PBS) containing 0.5% saponin (Sigma) at room temperature for 15 min. Then, the cells were stained with anti-IFN-γ (PE, clone XMG1.2; BioLegend) and anti-TNF-α (APC, clone MP6-XT22; BioLegend) at room temperature for 40 min. All data were collected by BD LSR Fortessa (BD Biosciences) and analyzed with FlowJo software (Tree Star Inc., Ashland, OR, USA).

### Competition ELISA Between RBD Protein and hDPP4 Receptor

Competition ELISA was performed to determine whether MERS-CoV antigen [RBD-[SSG]-FR, RBD-FR, RBD, and FR (negative control)]-immunized mouse serum inhibited binding of RBD protein to hDPP4 receptor (45, 46). 500 ng/well hDPP4 protein (Abcam) was coated on Nunc 96-well microtiter immunoplates (Thermo Fisher Scientific) and incubated overnight at 4°C. Plates were washed and blocked with 150 µl/well of blocking buffer [5% skim milk in PBS and Tween-20 (PBST)] for 1 h at 37°C. At the same time, mouse sera immunized with RBD, RBD-[SSG]-FR, RBD-FR, and FR were serially diluted (1/10 to 1/160) with 500 ng/well RBD protein (MERS-RBD-005P; eEnzyme) in TBST [50 mM Tris-Cl (pH 7.4), 0.05% Tween-20], added to new wells, and incubated for 1 h at 37°C. 100 µl solution was added to each well at 37°C and incubated for 2 h. After that, 100 µl of anti-6xHis tag antibody conjugated with horseradish peroxidase (1:1,000, Thermo Fisher Scientific) was added to each well and incubated for 1 h at 37°C. Plates were washed three times with TBST, and 100 µl/well of substrate TMB solution (BD Biosciences) was incubated at 37°C for 30 min in the dark. 50 µl of stop solution (2 N H_2_SO_4_) was added to the well to stop the color reaction and measure the absorbance at 450 nm using an ELISA reader FLUOstar OPTIMA (BMG LABTECH).

## Results

### The hRID Facilitated the Solubility of MERS-CoV RBD-FR

The spike glycoprotein (S) of MERS-CoV was used for the generation of MERS-CoV-like NPs. S protein forms trimers, resulting in large spikes on the virus envelope (47). It is challenging to express the full-sized S protein (~200 kDa) in *E. coli*. Thus, the S1 domain of S protein (~80 kDa), which includes the receptor-binding ability, was used. Our initial attempt to express the S1 domain, either as S1 or as an S1-FR fusion protein, failed; the expression level and solubility of the protein was below the lower limit of detection by SDS-PAGE and western blotting (Figure S1 in Supplementary Material). We therefore used the RBD (367–606 a.a.) of the S1 protein, which has a pivotal function as illustrated in Figure 1B (48, 49). When expressed alone in *E. coli*, the RBD is not able to form the trimeric assembly (unpublished observation), due to the lack of the HR2 domain within the S2 domain (50). To overcome this problem, FR was used as scaffold for the assembly. FR is a spherical NP whose subunits form trimers that subsequently result in octahedral structures composed of 24 identical subunits (51). We therefore performed computational modeling to evaluate the potential of FR as scaffold for trimer formation of the RBD.

Possible trimer formation was analyzed by computational modeling using MODELER (13, 52) and ClusPro (41, 42). Various linkers, including SSG, ASG, and D6, were introduced between the RBD and FR with a goal to minimize steric hindrance between the two domains so as to enhance trimer and NP formation. *In silico* analysis showed energy-stable trimeric models of RBD-FR, RBD-[SSG]-FR, and RBD-[ASG]-FR, whereas RBD-D6-FR failed to form a trimeric structure (Figure 1A). The RBD-[SSG]-FR was predicted to be the most stable and well-structured compared with RBD-FR and RBD-[ASG]-FR. Initial testing of the RBD-FR constructs without hRID fusion showed that none of the constructs were solubly expressed, even under low-temperature culture conditions (Figure 1C) (10.5 and 8.8%, for RBD-FR and RBD-[SSG]-FR, respectively). In addition, the yield of purified RBD-FR from a 2 l culture was only 30 µg of protein. Because of the low-soluble expression of MERS-CoV RBD, we fused hRID to the N-terminus of RBD-FR as a chaperna-based fusion partner (Figure 1B). We previously confirmed that by using chaperna, the globular domain of influenza hemagglutinin (HA) is efficiently assembled into a trimeric complex with an immunologically relevant conformation (Yang et al., *in press*). As shown in Figure 1C, the hRID fusion significantly increased the solubility of both RBD-FR (59.1%) and RBD-[SSG]-FR (62.83%), indicating that the chaperna platform effectively increased both the solubility and the folding of its fused target proteins. Because of the poor expression level and low solubility of the RBD-[ASG]-FR construct (Figure 1C), further experiments were performed using only the RBD-FR and RBD-[SSG]-FR constructs.

### The SSG Linker Increased the Proper Assembly of MERS-CoV RBD NPs

**Figure 2:**
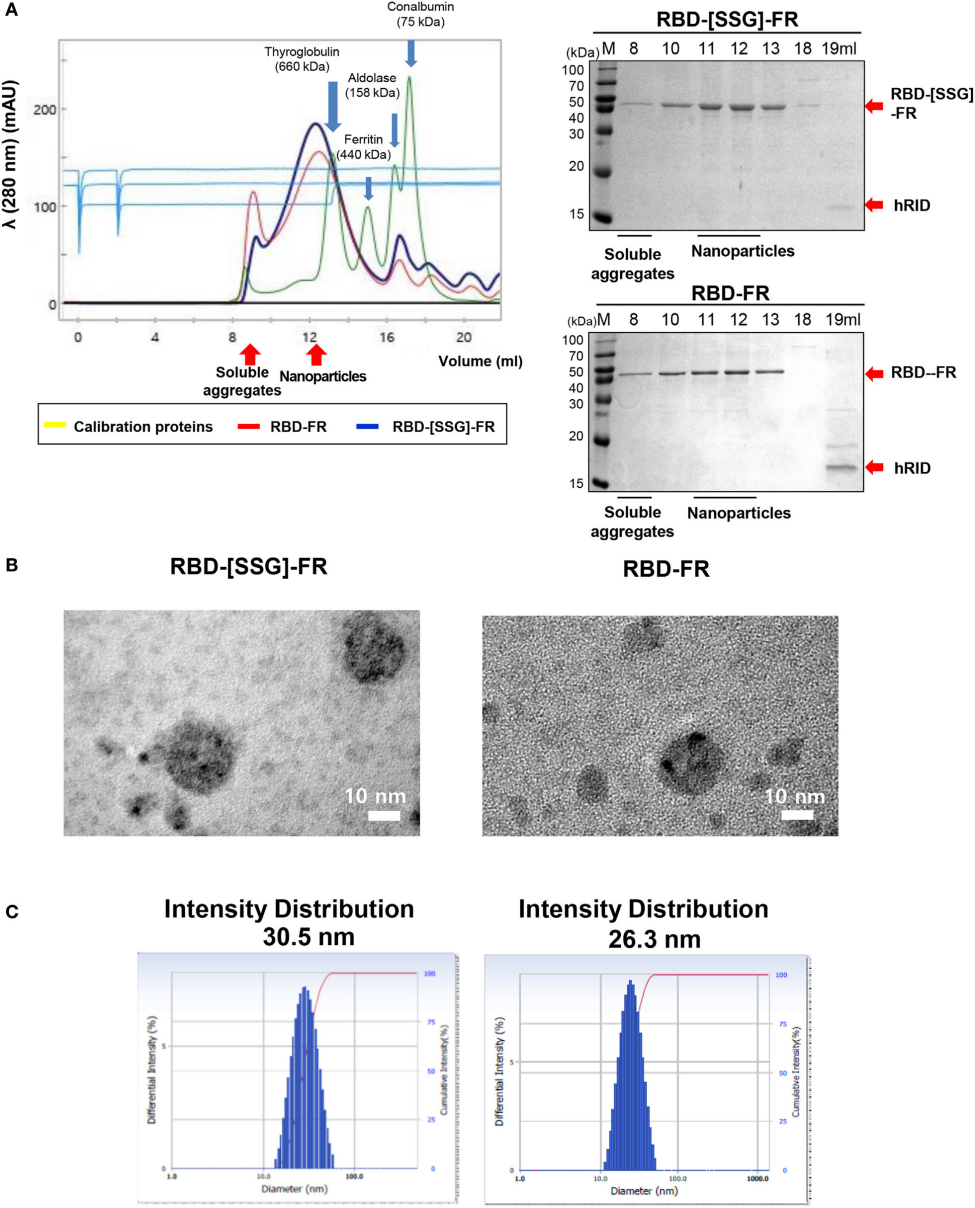
Analysis of Middle East respiratory syndrome-coronavirus receptor-binding domain (RBD) nanoparticle formation. **(A)** Size exclusion chromatography (SEC) of RBD-[SSG]-FR (blue) and RBD-FR (red) using Superose 6 Increase 10/300 GL. Blue arrows indicate the four eluted calibration proteins (yellow) with each size of the proteins were shown with the arrows. The eluted fractions were analyzed on sodium dodecyl sulfate-polyacrylamide gel electrophoresis (right panel). Red arrows indicate the purified target protein and the separated hRID. **(B)** Negative-stained transmission electron microscopy images of RBD-[SSG]-FR and RBD-FR. **(C)** The intensity distribution diameter of RBD-[SSG]-FR and RBD-FR as determined by dynamic light scattering.

After purification of the soluble proteins (Figure S2 in Supplementary Material), we determined the potential effects of using hRID as a fusion partner for the self-assembly of the NPs. As shown in Figure S3 in Supplementary Material, hRID-RBD-FR failed to form NPs. Because of this, we performed TEV protease cleavage of the hRID. Removal of the hRID domain facilitated the self-assembly of the RBD-FR monomers, and also eliminated the immune response against the non-self hRID domain in BALB/c mice (Figure S4 in Supplementary Material). After hRID cleavage, RBD-FR and RBD-[SSG]-FR were purified using SEC (Figure 2A). As expected, RBD-[SSG]-FR assembled into properly formed NPs (1,080 kDa) more efficiently than did RBD-FR NPs, which were mainly detected in the void-volume fractions, suggesting they were irregularly assembled soluble aggregates. The size of the RBD-[SSG]-FR NPs was further confirmed by TEM. TEM images of the RBD-[SSG]-FR NP structures showed hollow, spherical particles that were more compact than the RBD-FR NPs. The average diameter of the RBD-[SSG]-FR NPs was 28–30 nm (Figure 2B). In contrast, DLS analysis of the RBD-FR NP structure without the SSG linker appeared to be smaller with an average intensity diameter of 26.3 nm, and this compared with RBD-[SSG]-FR that had an average intensity distribution diameter of 30.5 nm (Figure 2C). Consistent with the SEC analysis, RBD-FR without a fusion partner was mostly produced in a soluble aggregated form. Therefore, we identified that the protein folding did not occur properly without hRID, and the formation of NPs was confirmed by both SEC and SDS-PAGE analyses. As shown in Figure S5 in Supplementary Material, the purified NPs retained their stability over an extended period of time at various temperatures (25, 4, and −20°C). Thus, these results indicate that the SSG linker allowed the RBD-FR to generate properly assembled NPs. It should also be noted that the efficiency of protein folding and NPs formation may be further enhance through appropriate linker selection.

### NaCl Concentration Had a Pivotal Role in Assembly of RBD-[SSG]-FR NPs

**Figure 3:**
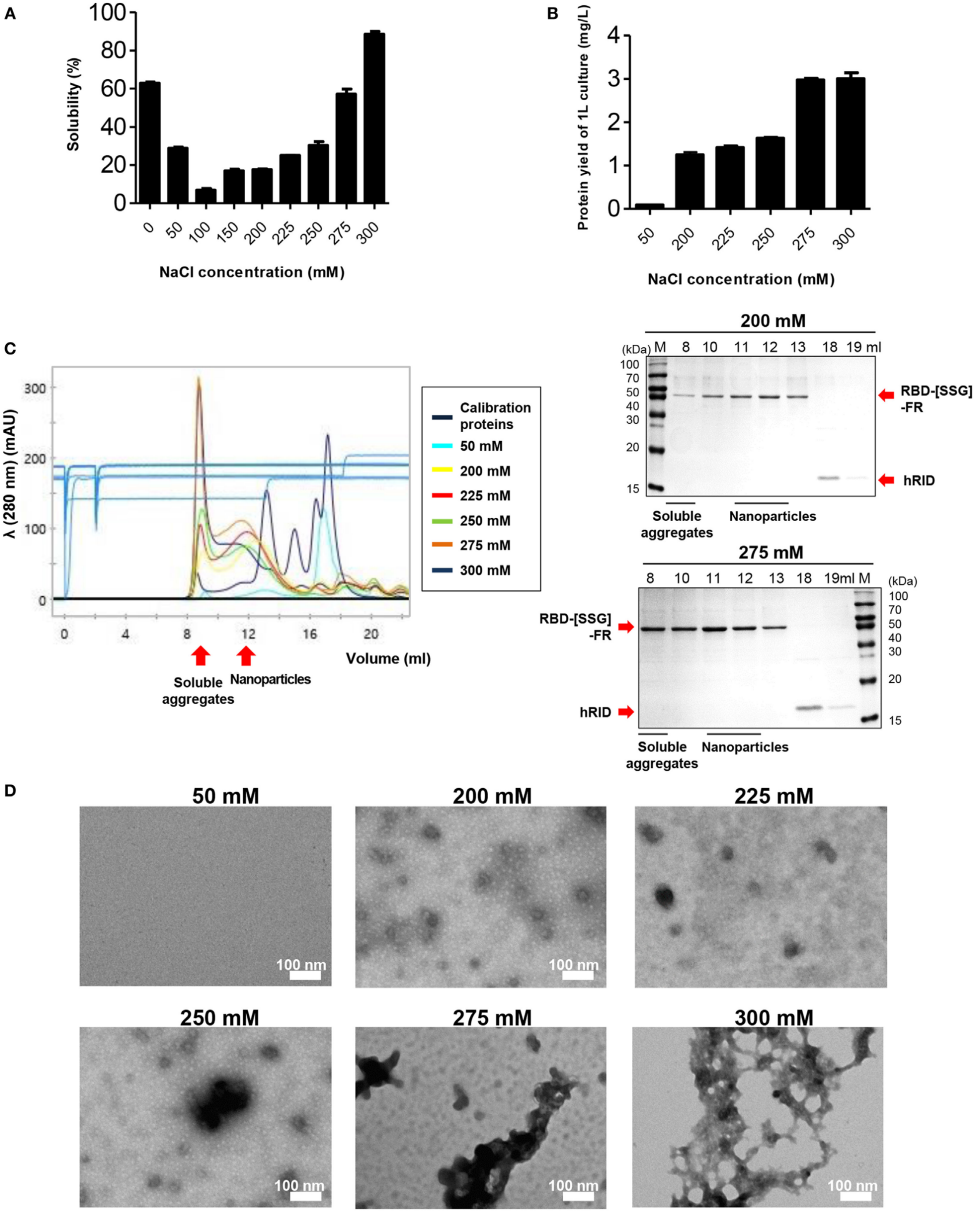
The effect of NaCl concentration on the assembly and stability of receptor-binding domain (RBD) nanoparticles (NPs). **(A)** Solubility of RBD-[SSG]-FR at various NaCl concentrations (0, 50, 100, 150, 200, 225, 250, 275, and 300 mM) was analyzed on sodium dodecyl sulfate-polyacrylamide gel electrophoresis and the data obtained from three independent experiments are summarized. **(B)** Purification yields (milligram per liter) of RBD-[SSG]-FR from 1-l cultures at various concentrations of NaCl (50, 200, 225, 250, 275, and 300 mM). The data are shown as the mean ± SD from duplicate experiments. The purified proteins from **(B)** were used for confirming NP formation of RBD-[SSG]-FR by size exclusion chromatography **(C)**, transmission electron microscopy **(D)**.

It has been reported that ionic strength plays an important role in the stability and self-assembly of ferritins (53, 54). We examined the effect of salt concentration on the formation and stability of the RBD-[SSG]-FR NPs at various concentrations (0–300 mM). Consistent with the previous studies, the stability of the protein was highly affected by the concentration of NaCl in the lysis buffer by SDS-PAGE (Figure 3A) (*n* = 3). The solubility of the protein significantly decreased as the concentration of NaCl increased from 0 to 100 mM, with the solubility being about 8.79-fold lower at 100 mM compared with 0 mM. Unlike previous studies, the solubility of the protein was gradually recovered at higher NaCl concentrations (>100 mM); the solubility at 300 mM was 1.45-fold higher than at 0 mM. Furthermore, the yield of soluble of protein per liter of culture increased in a salt concentration-dependent manner (Figure 3B).

To further investigate the effect of salt concentration, the physicochemical and morphological properties of the RBD-[SSG]-FR protein were examined by SEC, TEM, and DLS. In 50 mM NaCl, most of the protein was aggregated during the purification process, and the purified protein failed to form spherical structures, but instead, existed predominantly as 45 kDa monomers (Figures 3C,D; Figure S6 in Supplementary Material). In contrast, the protein that was lysed in 0 mM NaCl and purified in 200 mM NaCl, developed well-structured NPs according to TEM and DLS analyses (Figures 3C,D; Figure S6 in Supplementary Material). However, based on SEC analysis, at high-salt concentrations (>250 mM), the protein failed to form stable structures with the proteins being eluted predominantly in the void volume, suggesting they were soluble aggregates under the high-salt concentrations (Figure 3C).

Transmission electron microscopy images under the various salt concentrations clearly supported the conclusion, showing that the tendency for aggregation was dependent on the salt concentration (Figure 3D). Taken together, the results underscored the importance of salt concentration on the solubility of monomers and the quality of multimeric assembly of hybrid NPs.

### Fe^2+^ Had an Effect on the NPs Formation and Stability

**Figure 4:**
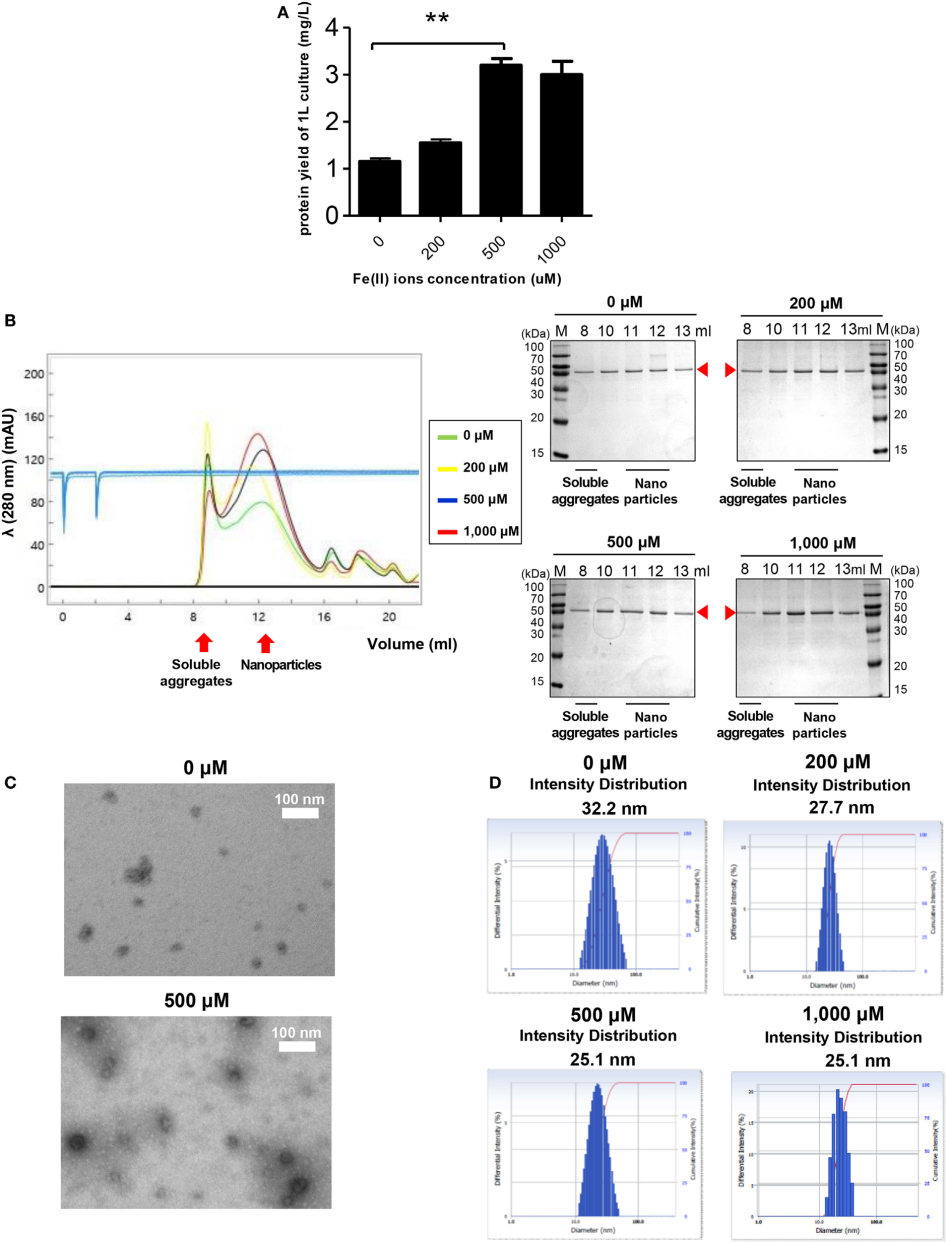
The effect of Fe^2+^ on the stability and nanoparticle (NP) formation of receptor-binding domain (RBD)-[SSG]-FR. **(A)** Purification yields of RBD-[SSG]-FR from cell culture at various concentrations of Fe^2+^ (0, 200, 500, and 1,000 mM). The data are presented as mean ± SD of duplicate experiments. All *p*-values were determined using Student’s two-tailed tests (**p < 0.01). After purification, the NPs were examined by size exclusion chromatography **(B)**, transmission electron microscopy **(C)**, and dynamic light scattering **(D)**. Arrowheads in **(B)** indicate the eluted RBD-[SSG]-FR.

Ferritin has an intrinsic ability to interact with Fe^2+^ to form ferritin-iron cores (55). Thus, it was worth investigating the effect of Fe^2+^ on the assembly and stability of RBD-[SSG]-FR NPs. Cells were grown in LB medium with various concentrations of Fe^2+^. As shown in Figure 4A, the yield of purified protein was significantly increased from cultures with 500 µM Fe^2+^, reflecting a 2.7-fold increase compared with similar cultures 0 µM Fe^2+^. The cell growth and purification yield at 1,000 µM Fe^2+^ were slightly decreased, presumably due to the toxicity of ferric acid. NP formation under the various concentrations of Fe^2+^ was analyzed by SEC (Figure 4B). Consistent with the previous results, the proteins were eluted mainly in the fractions expected for the size of assembled NPs (1,080 kDa).

Of note, the ratio between NPs and soluble aggregates in the SEC analysis showed that NP formation was facilitated at high concentrations of Fe^2+^ (Figure 4B). The formation of RBD-[SSG]-FR NPs at an Fe^2+^ concentration of 1,000 µM was confirmed by TEM (Figure 4C) and DLS (Figure 4D). The TEM analysis clearly showed that the morphology of the proteins was more compact, and probably highly stable, when assembled at high Fe^2+^ concentrations (500 µM) than at lower concentrations (0 µM) (Figure 4C). As shown in Figure 4D, the average diameter of NPs examined by DLS was 25.1 nm at high Fe^2+^ concentration (500–1,000 µM) and 27.7–32.2 nm at lower concentration (0–200 µM). These results suggest that both Fe^2+^ and salts concentrations influenced the efficiency and quality of the regular assembly of hybrid ferritin monomers into NPs.

### RNA Binding Was a Key Factor for the Solubility of Hybrid Ferritin

**Figure 5:**
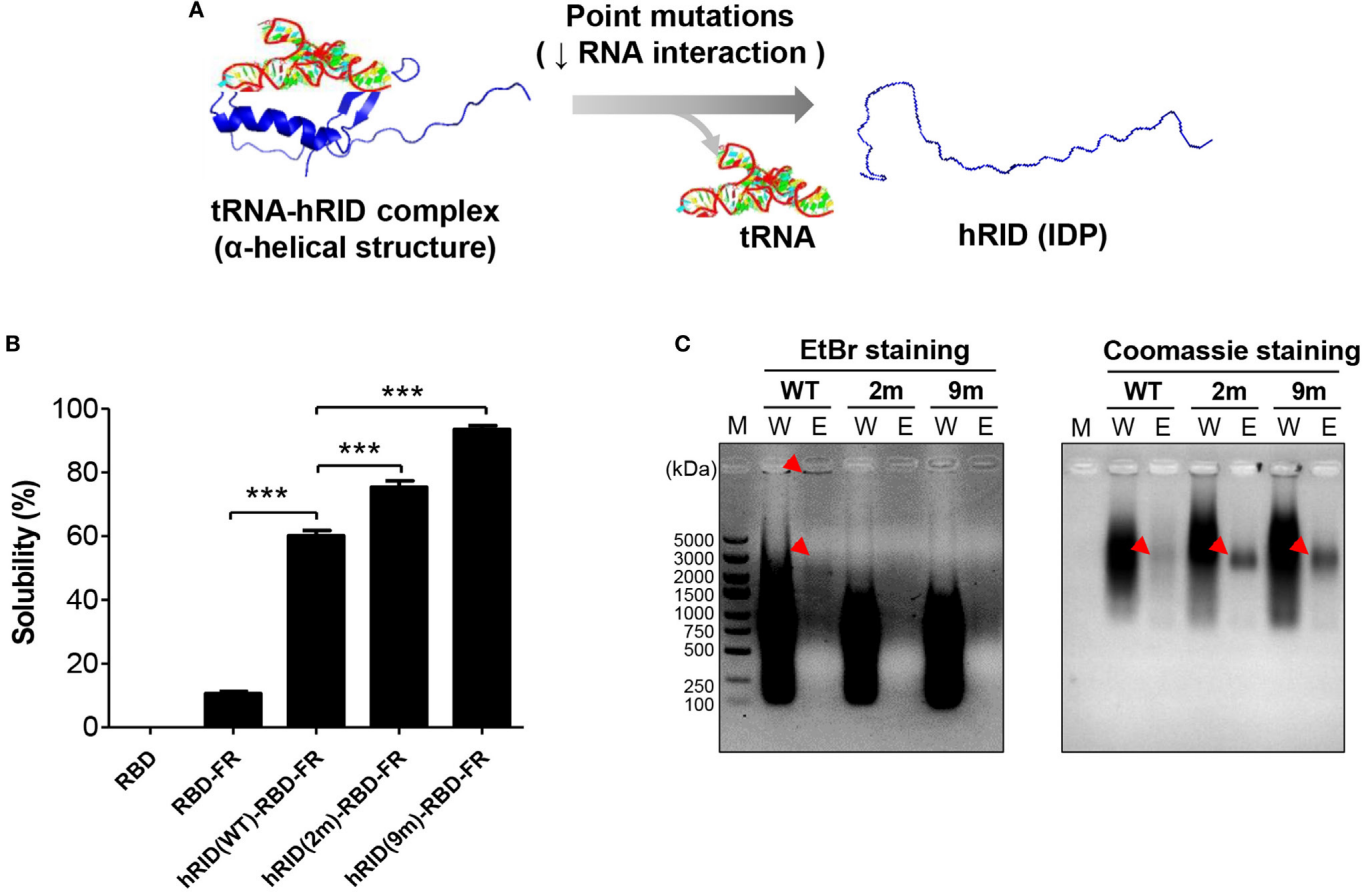
Verifying the role of RNA in protein solubility and stability. **(A)** Schematic illustration of the hRID structure with and without tRNA binding. Mutant hRID with impaired tRNA binding failed to form the specific structure and remained disordered, whereas wild-type hRID interacted with tRNA and formed α-helical structures. **(B)** The solubility of receptor-binding domain (RBD), RBD-FR, hRID(WT)-RBD-FR, hRID(2 m)-RBD-FR, and hRID(9 m)-RBD-FR was analyzed on sodium dodecyl sulfate-polyacrylamide gel electrophoresis and summarized. The data obtained from three independent experiments were used (***p < 0.001). **(C)** Identification of RNAs co-purified with hRID(WT, 2 m, or 9 m)-RBD-FR. The arrowheads on the ethidium bromide-stained gel and coomassie blue-stained gel represent the co-purified RNAs and purified proteins, respectively. The molecular weight marker (M), wash fraction (W), and elution fraction (E), are indicated. Each soluble fraction from cell lysates was purified using Hispur™ Ni-NTA Resin.

Our previous studies show that an RNA–protein interaction is crucial for transducing the chaperone function of RNA into the folding of client proteins (38). Consistent with that, our present study showed that RNA facilitated the folding of its interacting proteins. The solubility of hRID(WT)-RBD-FR was 5.69-fold higher than RBD-FR without hRID fusion (Figure 1), strongly supporting the previous studies. In addition, the solubility of RBD alone was completely insoluble (Figure 5B; Figure S7 in Supplementary Material). It has been shown that the positively charged residues of lysine moieties in hRID contribute to tRNA binding (56). In the current study, the tRNA binding induced the intrinsically disordered protein (IDP) status of hRID to form alpha-helical structures (Figure 5A). Thus, two RNA-binding mutants, double-mutant hRID (K19A and K23A) and nona-mutant hRID (K19A, K23A, R24A, K27A, K30A, K31A, K35A, K38A, and K40A) were constructed (Table S1 in Supplementary Material). The total *E. coli* lysate (T) was fractionated into the soluble fraction (S) and the pellet fraction (P) by centrifugation. As expected, both RBD and RBD-FR without fusion to hRID domain, were refractory to being produced as soluble proteins (Figure 5B). Interestingly, the solubility of the RNA-binding mutants did not decrease, but actually increased to 75.3% for the 2 m mutant and 93.4% for the 9 m mutant compared with wild-type protein at 60.1% (Figure 5B). Considering that hRID is relatively unstructured in the absence of tRNA binding, the results are consistent with previous reports that the fusion with IDPs promotes the solubility of target proteins (57–59). Following purification of wild-type hRID-RBD-FR (hRID(WT)-RBD-FR), electrophoretic mobility shift assays showed that greater amounts of nucleic acids were co-purified with hRID(WT)-RBD-FR protein than with the mutant hRID-RBD-FRs (2 and 9 m) under non-denaturing conditions (Figure 5C). The relative ratio of nucleic acid based on EtBr staining and proteins based on Coomassie staining in the eluted fraction confirmed the reduced affinity of mutants to nucleic acids.

**Figure 6:**
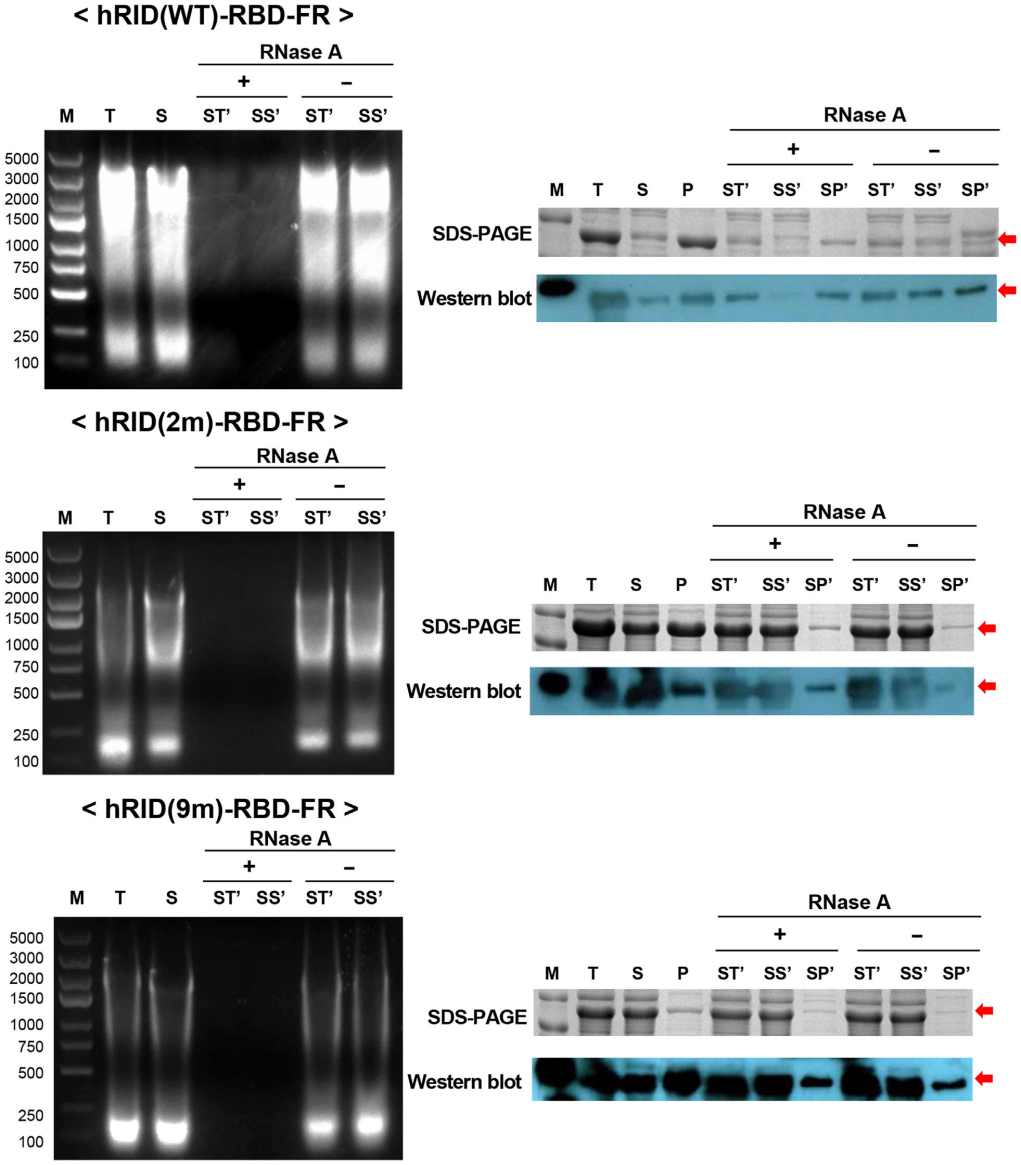
RNA’s function to maintain protein stability. The effect of RNase A treatment on the solubility of hRID(WT, 2, and 9 m)-receptor-binding domain (RBD)-FR. RNA elimination by RNase A was analyzed on an agarose gel (left). Following RNase A treatment, the soluble fraction (S) was further separated into total (ST′), soluble (SS′), and insoluble (SP′) fractions and analyzed by sodium dodecyl sulfate-polyacrylamide gel electrophoresis (SDS-PAGE) (right, upper panel) and western blot (right, lower panel).

To test if RNA had a role in maintaining the stability of the target proteins, the lysates were treated with RNase A to eliminate RNA, and the solubility of each protein was analyzed by SDS-PAGE and western blotting. The soluble fractions of the lysates (S) were incubated at 37°C in the presence and absence of RNase A and the samples were further separated into soluble fraction (SS′) and insoluble fraction (SP′) by centrifugation. As shown in the left panel of Figure 6, RNase A treatment completely abolished the effect of RNA on protein solubility as compared with the control (RNase A−) or with samples prior to RNase treatment. Parallel experiments with the 2 and 9 m mutants showed much less RNA co-purified with the proteins, confirming the reduced affinity to nucleic acids and the complete depletion of RNA by RNase A treatment (Figure 6, left panel). Remarkably, the solubility of hRID(WT)-RBD-FR was greatly reduced by depletion of RNA as reflected in the ratio of [SS′]/[SP′] [0.1 and 0.4 for RNase (+) and RNase (−), respectively] by both Coomassie staining and western blot analyses (Figure 6, right panel). However, the solubility of the mutants (2 and 9 m), was not significantly affected by RNase A treatment, probably due to their lower affinity to RNA (Figure 6, right panel). Taken together, the results demonstrate that hRID(WT)-RBD-FR maintained a strong affinity for RNA, and that affinity was pivotal for maintaining the solubility of the protein.

### RNA-Binding Facilitated NP Assembly and Prevented Formation of Soluble Aggregates

**Figure 7:**
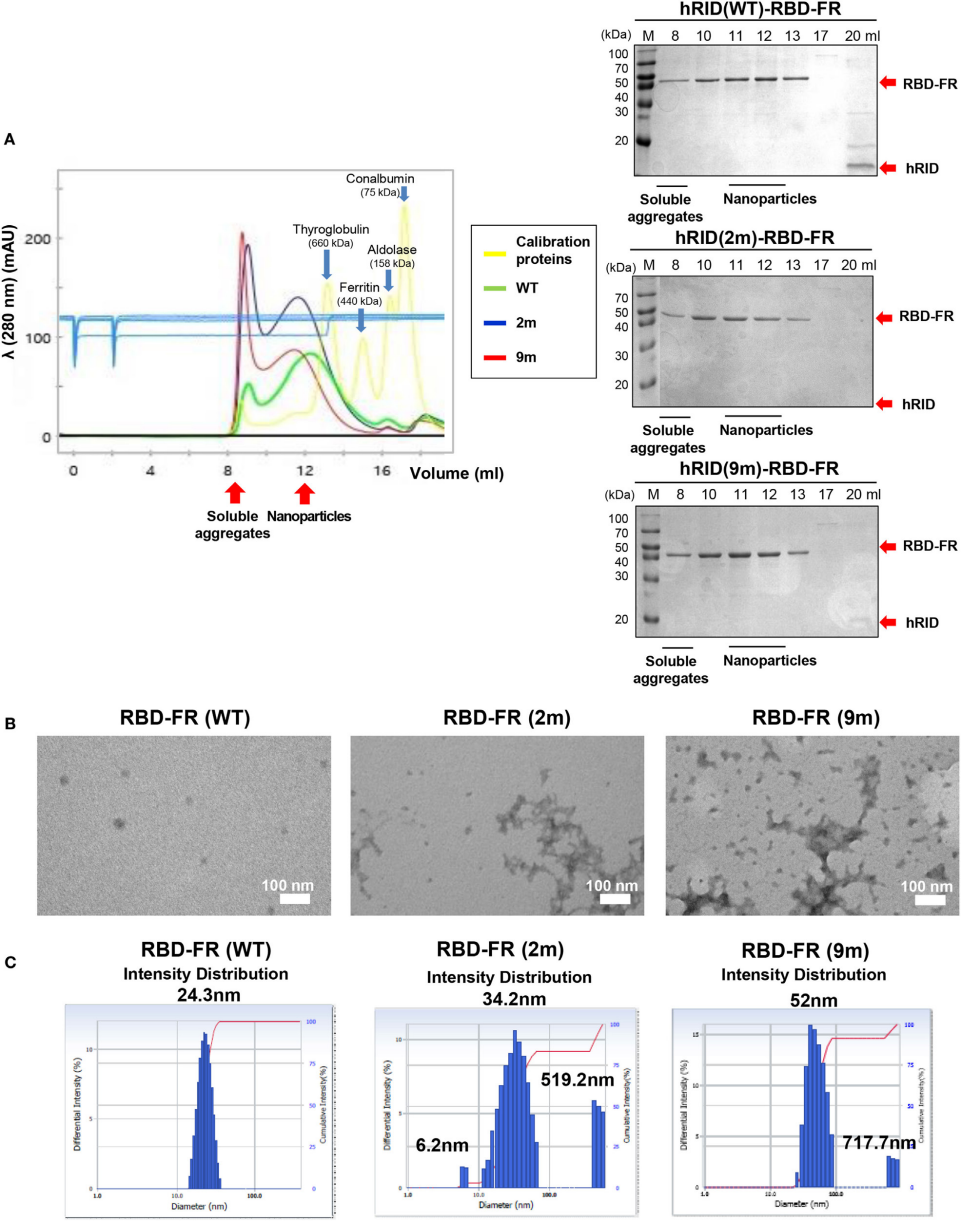
Elucidation of RNA-mediated nanoparticle (NP) formation of receptor-binding domain (RBD)-FR. **(A)** Size exclusion chromatography analysis of RBD-FR NPs purified from the TEV protease-cleaved hRID(WT, 2, or 9 m)-RBD-FR. The fractions (11–12 ml) estimated as NPs were further analyzed by transmission Electron Microscopy **(B)** and dynamic light scattering **(C)**.

To further define the RNA dependence of solubility of the ferritin hybrids (Figure 6), we investigated if the RNA binding had a role in the formation of NPs. RBD-FR and the various hRID-RBD-FR (WT, 2, and 9 m) proteins were purified by nickel-affinity chromatography (Figure S2 in Supplementary Material) and their physicochemical properties analyzed by SEC (Figure 7A), TEM (Figure 7B), and DLS (Figure 7C). The soluble yields of RBD-FR (hRID fusion) was approximately 1.6 mg/l of culture, representing greater than 1,000-fold higher levels than its hRID (−) counterpart (~15 μg/l culture), again confirming the role of hRID as a robust enhancer for solubility and assembly. It was striking to note that the two mutant proteins, despite high solubility (Figure 5B), were detected at disproportionately higher amounts in the void fractions of SEC, indicating that they failed to form NPs of a defined size, and existed predominantly as soluble aggregates (Figure 7A). However, hRID(WT)-RBD-FR predominantly formed NPs of a defined size (~1,080 kDa). It is also interesting to note that there was a slight shift of the RNA-binding mutants (2 and 9 m) in the elution pattern, suggesting a larger size of NPs compared with wild-type NPs. Overall, the ratio between soluble aggregates in the void volume and the NPs of defined size clearly showed that RNA binding was crucial for assembly of the monomers into NPs. As a control, RBD-FR (without hRID fusion) existed predominantly as soluble aggregates (Figure S8 in Supplementary Material). Consistent with these results, EM analysis confirmed well-structured NPs by hRID(WT)-RBD-FR, compared to largely aggregated structures by the mutant proteins (Figure 7B). Even if multi-molecular structure was formed, the structure becomes unstable, mostly as soluble aggregates. Consistently, the intensity distribution diameter of the wild-type protein, as estimated by DLS analysis, was 25 nm compared with larger sizes of hRID(2 m) at 34.2, 519.2 nm and hRID(9 m) at 52, 717.7 nm (Figure 7C; Figure S9 in Supplementary Material). It is conceivable that soluble aggregates may shield the exposed 6-histidine tag, resulting in a decreased binding affinity to Nickel resins and elution in earlier fractions compared with WT protein (Figure S2 in Supplementary Material). Taken together, the data demonstrate that RNA binding prevented aggregation into irregular conformations and guided the self-assembly of the hybrid ferritin monomers into NPs of a stable structure.

### Wild-Type Proteins Showed Strong-Binding Ability to Both the hDPP4 Receptor Protein and MERS-CoV Infected Patient Sera

**Figure 8:**
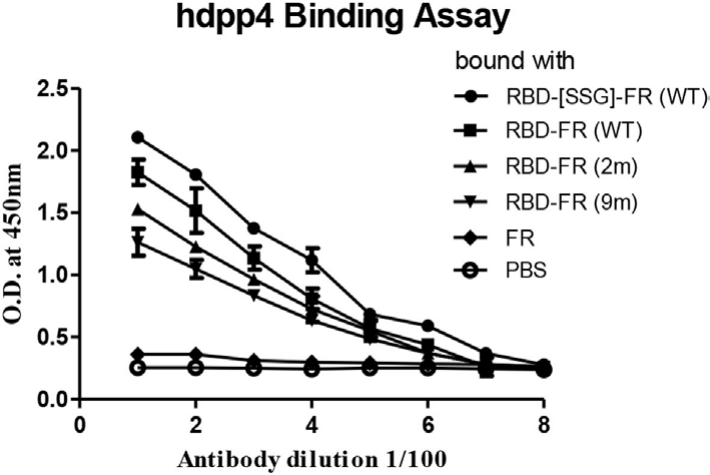
Enzyme-linked immunosorbent assay analysis of receptor-binding domain (RBD)-FR bound with hDPP4 receptor protein. Each purified protein shown in Figure S2 in Supplementary Material and size exclusion chromatography was used to explore hDPP4 receptor-binding affinity to the protein. All data are shown as mean ± SD from triplicate samples. FR alone and phosphate-buffered saline were used as negative controls.

**Figure 9:**
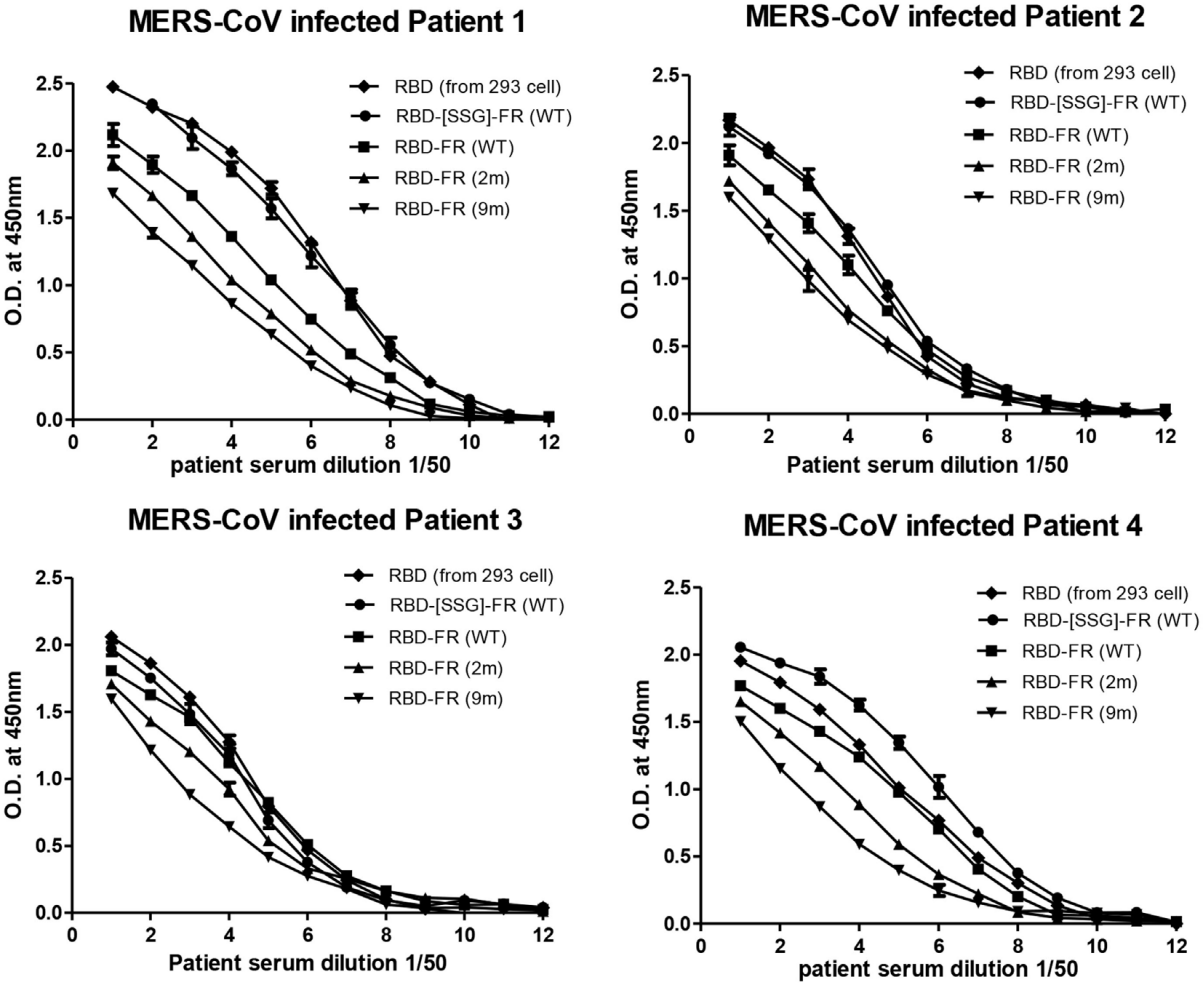
Sero-diagnosis of Middle East respiratory syndrome-coronavirus-infected patients using receptor-binding domain (RBD)-FR nanoparticles. The purified proteins shown in Figure S2 in Supplementary Material were used as coating antigens. FR alone and infected cell lysates were used as negative and positive controls, respectively. Virus-infected sera from four patients were serially diluted from 1:100 (twofold dilution). All data are presented as mean ± SD of duplicate samples.

The immunological properties of ferritin NPs were analyzed by ELISA. The hDDP4 (human DPP4) receptor has been previously identified as the receptor for MERS-CoV human infection (46). Therefore, using hDPP4 as a coating antigen, ELISA-binding assays between RBD NPs and the receptor were performed (Figure 8). FR without RBD fusion failed to bind, and was similar to the PBS negative control. Strikingly, the binding ability increased in the same order as the RNA-binding ability (hRID(WT) > 2 m > 9 m), with highest absorbance observed in the WT with the SSG linker (hRID(WT)-RBD-[SSG]-FR). The results show that the conformation of RBD in the WT NPs better resembled the protective antigen of MERS-CoV RBD from 293 cells, compared with the RNA-binding mutants 2 and 9 m. Again, judicious choice of linker between the ferritin carrier and the antigen was important for receptor binding and was reflected in its importance for NP assembly into a stable conformation (Figure 2). Finally, the ELISA results for NP against human patients was investigated using the sera from four MERS-CoV-infected patients (Figure 9). Six different proteins, including five recombinant NPs (hRID(WT)-RBD-FR, hRID(2 m)-RBD-FR, hRID(9 m)-RBD-FR, hRID(WT)-RBD-[SSG]-FR, and FR), and MERS-CoV RBD protein were compared by ELISA using them as capture antigens. Strong ELISA signals were detected for the four recombinant NPs and MERS-CoV RBD from 293 cells (positive control). The WT form consistently showed a higher response than the RNA-binding mutants (hRID(WT) > 2 m > 9 m), with hRID(WT)-RBD-[SSG]-FR being the best binder among constructs tested. These results address to the utility of the *E. coli* assembled MERS-CoV RBD-FR NPs as useful tools for sero-diagnosis of MERS-CoV infection. Taken together, the results confirmed the immunologically relevant conformation of the MERS-CoV RBD displayed on the hybrid ferritin particles, and the crucial role of RNA in controlling the kinetic pathway for the assembly of viral antigen monomers into stable NPs.

### Immunization of MERS-CoV NPs Induces RBD-Specific Antibodies

**Figure 10:**
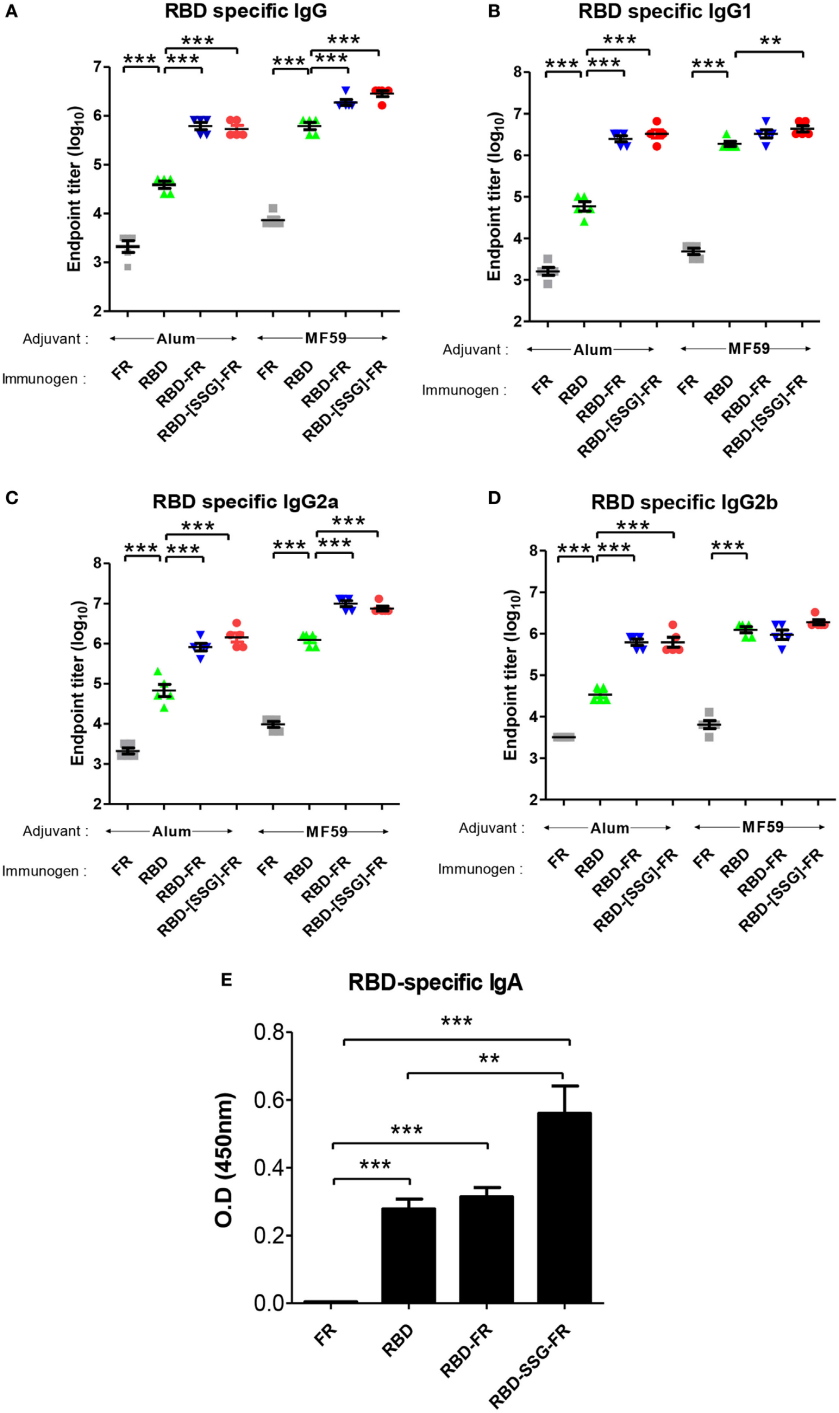
Immune responses in receptor-binding domain (RBD) nanoparticles (NPs) immunized mice (*n* = 5). Endpoint titer of IgG **(A)**, IgG1 **(B)**, IgG2a **(C)**, and IgG2b **(D)** antibody binding to Middle East respiratory syndrome-coronavirus RBD were detected using mice serum after two immunizations. RBD-specific antibodies were detected after immunizations of RBD NPs, RBD, FR with adjuvant (alum and MF59) using enzyme-linked immunosorbent assay. **(E)**. RBD-specific IgA antibodies were detected using BALF (diluted 1:8) after immunization of protein with MF59. OD, optical density. Each endpoint titer was shown by individual. All error bars were shown as mean ± SD (*n* = 5) and all *p*-values were obtained using Student’s two-tailed tests (**p < 0.01, ***p < 0.001).

To evaluate the immunogenicity of ferritin-based NPs, BALB/c mice (*n* = 5) were immunized with RBD, RBD-FR, and RBD-[SSG]-FR NPs antigens. The tRNAs were found to be removed from the hRID protein during the purification process. Before immunization, potential RNA contamination in the purified proteins was determined by gel electrophoresis. As shown in Figure S11 in Supplementary Material, RNA was below detection level, if any, after several purification steps, compared with the proteins purified in the first step. Previously, MF59-adjuvated and alum-adjuvated MERS-CoV antigen have been reported to increase the antibody and T-cell responses in mice (44, 60). Thus, the first group and second group were immunized twice with 20.0 µg of antigen containing the equal volume of alum and MF59, respectively (Figure S10 in Supplementary Material). After collecting serum, the RBD-specific IgG titer was analyzed by ELISA. Alum adjuvanted RBD-[SSG]-FR NPs and RBD-FR NPs induced 14-fold and 16-fold higher (*p* < 0.0001) than RBD (Figure 10A). The antibody titers of MF59 adjuvanted RBD-[SSG]-FR NPs and RBD-FR NPs were 4.5- and 3-fold higher than RBD, respectively. The antibody responses by RBD-FR and RBD-[SSG]-FR NPs were much stronger than the RBD in all antibody subtypes tested (IgG1, IgG2a, and IgG2b) (Figures 10B–D). As a test of mucosal immune responses, the RBD-specific IgA antibody levels from BALF were also analyzed by ELISA (Figure 10E). MF59 adjuvanted RBD-[SSG]-FR NPs presented significantly higher OD values than RBD and FR (negative control). These results suggested that RBD-[SSG]-FR NPs induces local mucosal immune response stronger than RBD. In addition, it was confirmed that antibody responses of IgG, IgG1 (Th1), IgG2a, and IgG2b (Th2) against MF59-adjuvated antigens were higher than those from alum-adjuvated antigens. In contrast, PBS and FR control groups failed to, or only weakly induce an antibody response against RBD protein. These results suggest that FR-based NPs significantly enhance various antibody responses than monomeric antigens.

### Specific Cellular Immune Responses Were Induced by MERS-CoV NPs

The cellular immune responses were investigated in mice immunized with protein (RBD, RBD-FR, RBD-[SSG]-FR) and FR (negative control). Splenocytes of mice (*n* = 4) were harvested 1 week after the last immunization, stimulated with RBD protein, and analyzed for cytokines by flow cytometry. In the RBD-immunized group, IFN-γ and TNF-α-producing CD4^+^ T-cell responses were detected at low levels. However, IFN-γ and TNF-α-producing CD4^+^ T cells were significantly increased in RBD-FR and RBD-[SSG]-FR-immunized groups compared with RBD and FR-immunized group (Figure S12 in Supplementary Material). These results demonstrated that the RBD NPs vaccination induced antigen-specific CD4^+^ T cells that produced IFN-γ and TNF-α upon antigen stimulation.

### Anti-NPs Serum Effectively Blocked RBD Protein Binding to the hDPP4 Receptor

**Figure 11:**
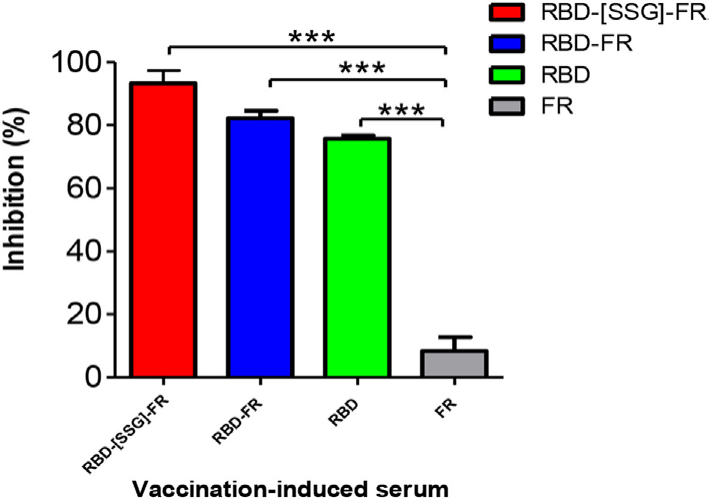
Nanoparticles-immunized mouse serum inhibited interaction between Middle East respiratory syndrome-coronavirus receptor-binding domain (RBD) and hDPP4 receptor. Competition enzyme-linked immunosorbent assay showed that anti-RBD mouse sera (1:10, from mice immunized with RBD-[SSG]-FR, RBD-FR, and RBD) blocked binding between RBD (5 µg/ml) and hDPP4 receptor (5 µg/ml). FR-immunized mouse serum (1:10) was used as a negative control. All sera were serially diluted from 1:10 (twofold dilution). All data are presented as mean ± SD (*n* = 5) and *p*-values were obtained using Student’s two-tailed tests (***p < 0.001).

Middle East respiratory syndrome-coronavirus infection is mediated by the interaction of RBD and the host receptor hDPP4 (45, 46). As a correlate of protection, a competition ELISA was performed to investigate whether antibodies generated from NPs immunization were able to interfere with the binding to hDPP4. Thus, after incubation of RBD protein with mouse serum (1:10), the binding of serum-mixed samples to hDPP4 protein was measured. As shown in Figure 11, RBD-[SSG]-FR, RBD-FR, and RBD-immunized sera strongly abolished the binding of RBD to hDPP4 receptor (93.3, 82.2, and 75.67%, respectively). Interestingly, the relative efficiency of interference correlates with that of NP assemblage (Figure 11). In contrast, the FR-immunized mouse serum (negative control) failed to inhibit the interaction. Taking together, these results demonstrate that immunization of NPs greatly stimulates MERS-CoV-specific antibody response that effectively interferes with the cellular receptor binding, suggesting its possibility as a vaccine. However, protection efficacy should ultimately be tested in a live virus challenge model.

## Discussion

Having key immunologic features, like a highly repetitive nanostructure, provides a designing principle for NPs in inducing potent and long-lasting antibody responses. For VLPs of non-enveloped viruses, assembly is made purely by capsid proteins. For enveloped viruses, however, additional membrane components and matrix proteins are required to display the target antigens on the surface of assembled VLPs. A promising alternative is to present target antigen on the surfaces of self-assembled NPs, which, in lieu of lipid membranes and matrix proteins, serve as a macromolecular scaffold for the presentation of antigens of interest (61). Ferritins, as a substitute for matrix proteins and membranes, have been used as scaffold for the regular assembly of target antigens. However, ferritin-based NPs have been produced only in host cells of mammalian or insect origin (28, 62). Previously, we showed that influenza HA could be assembled in a soluble, trimeric, and immunologically relevant conformation by exploiting chaperna activity (63). The present study is the first report of using RNAs as molecular chaperone for supra-molecular structures. Here, we present a novel bacterial system for NP assembly of hybrid ferritin displayed surface antigens from MERS-CoV. The NPs reacted strongly with sera derived from MERS-CoV-infected patients (Figure 9) confirming their utility in sero-diagnosis of infection. Moreover, the antisera, generated from immunization of mice, were able to interfere with the binding to the cellular receptor hDPP4 (Figure 8), in part of essential protective immune responses. The efficiency of receptor-binding inhibition (Figure 11), as well as the ability for inducing the mucosal responses (Figure 10E), correlated with the regular assembly of NPs as examined by DLS or EM (Figure 2), confirming that presentation of antigenic epitopes on a multivalent and highly repetitive structure is indeed important for the quality of immune responses. Overall, the quality of NPs and consequent immune responses were governed by the RNA-mediated assembly of antigens.

We hypothesized that chaperna function could be harnessed for presenting target antigens as highly repetitive nanostructures (Figure 1D). The hRID is the N-terminal domain of hLysRS and was previously identified as a nucleic acid-binding domain (Figure 5) (63). In this report, the hRID was exploited as a transducer for chaperna function (TCF) by serving as a docking-tag for cellular RNA for the folding/assembly of the hybrid FR containing client antigen proteins [RBD of MERS-CoV (Figure 1D)]. The advantage of using hRID as a TCF could be many fold. First, hRID is small (8.3 kDa), monomeric, and was flexible enough to allow the access of site-specific protease for the removal of hRID (Figure S3 in Supplementary Material). Of note, hRID belongs to IDPs, which switches into stabile α-helixes upon binding with tRNAs. Second, the bound RNA, due to its highly negative charge, may resist uncontrolled intermolecular interactions among monomers into amorphous aggregation. Finally, even the naked hRID (in the absence of RNA binding), due to its intrinsically flexible nature, may not pose physical hindrance to multiple interactions among monomers, enabling assembly into stable super-structures, upon removal of the hRID. Thus, the potential “pace-making” function harnessed with the RNA molecule, allows a regular assembly of monomers as highly repetitive nanostructures. Consequently, in the current study, hybrid FR was produced in soluble forms, could be purified by one-step affinity chromatography, and most remarkably, assembled into NPs of defined sizes upon removal of the hRID (Figure S2 in Supplementary Material). Consistent with the principles of design, the loss of RNA binding by hRID significantly hampered the regular assembly of the ferritin monomers and increased the amount of non-functional misfolded proteins as soluble aggregates (Figure 7). Thus, the overall yield, as well as the quality of NPs, were dependent on the chaperna function transduced by the hRID, which in turn was mediated by interaction with cellular RNAs (likely to be tRNAs).

The driving and controlling factors for *de novo* assembly of biomolecules are poorly understood. Historically, host factors like GroEL/S were initially discovered as molecular chaperones for supporting viral growth in *E. coli* and supporting the assembly of viral capsid proteins (64, 65). Moreover, GroEL/S also cooperates with RbcX in plant cells for the assembly of multi-component RUBISCO, which is the most abundant protein in the biosphere responsible for photosynthesis (66). Therefore, it is intriguing that RNA could provide such a robust folding/assembly of a supra-molecular structure. We recently confirmed that the present strategy could be successfully applied to the assembly of bacterially synthesized monomers of norovirus into VLPs composed of 180 monomers (unpublished observation, Seong, B.L.). Whether RNA can substitute for, or collaborate with pre-existing protein-based molecular chaperones remains an exciting avenue for future investigations. It should be noted that the defined versatile functions are being expanded for RNA molecules. As an engineered system for harnessing chaperna function, the present report may prove to be the tip of an iceberg for pivotal function of RNA molecules as chaperones for the folding and supra-molecular assembly of proteins in living organisms (36, 38).

Various factors were identified as important for efficient assembly of MERS-CoV NPs. As an extrinsic factor, the binding affinity of hRID to cellular RNAs was crucial for the assembly and the quality of the assembled NPs (Figure 7). As intrinsic factors, the concentration of salts and Fe^2+^ also influenced the assembly and stability of NPs (Figures 3 and 4). The ionic strength played an important role in the stability and self-assembly of ferritins, and aggregation increased with increasing concentrations of NaCl (54). The assembly of the hybrid MERS-CoV NPs revealed an interesting change in salt dependence, with 200–225 mM NaCl buffer as optimal condition as confirmed by EM and DLS analyses (Figure 3). The change in salt dependence was probably due to the presence of electrostatic interactions among RBD domains (54, 67). The dependence on Fe^2+^ was not surprising considering that ferritin has an intrinsic ability to interact with Fe^2+^ to form ferritin-iron cores (55). Based on our experience, to enhance the quality of NPs, it is advisable to control Fe^2+^ concentrations, both during the culturing of the bacterial cells and during the purification of the soluble monomer proteins (Figure 4). First, the yield of the purified protein was increased in the presence of 500 µM Fe^2+^ (Figure 4B), up to 2.7-fold greater compared with the control conditions lacking Fe^2+^. Second, the ratio between NPs and soluble aggregates in SEC showed that NPs formation was facilitated at high concentrations of Fe^2+^, and resulted in a more compact morphology under EM (Figures 4B,C). Thus, both the overall yield and the quality of NPs were governed by their intrinsic ability to interact with Fe^2+^. Finally, our data show that the presence and the nature of the linker between the ferritin and the RBD antigen was also important to the assembly of NPs. It is possible that a linker with flexibility and sufficient length would accommodate the steric requirements for assembly of multimeric NPs. However, it is difficult to precisely predict the effect of the linker, and therefore it is advisable to screen multiple constructs during the early stages of testing the assembly of NPs displaying antigens of interest.

In conclusion, the chaperna-based antigen assembly platform holds promise for the development and delivery of NP-based vaccines to enhance RBD-specific antibody responses, and the serological detection of emerging viruses. Various types of designing principles have advanced the structure-based approaches to NP assembly (61, 68). However, most of the *in silico* methods consider the thermodynamic stability of the final assembled NPs, but not necessarily the kinetic pathways leading to their successful folding into regular assemblages. Consequently, most NPs are refractory to soluble expression and fail to assemble as designed, resulting in significant, and practical challenges in the manufacturing process. The chaperna-mediated folding and the “pace-keeping” assembly of monomers into higher ordered structures will enable faithful production of NP and VLP-based vaccines against emerging and re-emerging viral infections.

## Ethics Statement

This study was carried out in accordance with the recommendations of “Ministry of Food and Drug Safety (MFDS) of Republic of Korea.” The protocol was approved by the “YLARC Institutional Animal Care and Use Committee (IACUC; permit number: IACUC-A-201710-377-01).”

## Author Contributions

Y-SK and BS designed the research study. Y-SK, AS, and BS wrote the manuscript. Y-SK performed overall experiments. JHK, JEK, SK, YB, and JS conducted the research study. JL and JY provided technical assistance and vector. PK developed the necessary software for performing analyzing computer data. CP provide technical assistance to conduct RNase A treatment experiments. YK and NC provide patients sera infected with MERS-CoV. MK and JC analyzed cellular immune response by flow cytometry.

## Conflict of Interest Statement

The authors declare that the research was conducted in the absence of any commercial or financial relationships that could be construed as a potential conflict of interest.
