# Supplementary material for: Chaperna-Mediated Assembly of Ferritin-Based Middle East Respiratory Syndrome-Coronavirus Nanoparticles
Source: Front Immunol. 2018 May 17;9:1093. doi: 10.3389/fimmu.2018.01093 (PMC5966535; doi:10.3389/fimmu.2018.01093)
Supplement: Supplementary file 1 [file presentation_1.PDF]

Supplementary Material

Chaperna-mediated Assembly of Ferritin-based MERS-CoV Nanoparticles

Young-Seok Kim[1,2], Ahyun Son[1], Jihoon Kim[1,2], Soon Bin Kwon[1,2], Myung Hee Kim[3], Paul Kim[1,2], Jieun Kim[6], Young Ho Byun[1], Jemin Sung[1,2], Jinhee Lee[1,2], Ji Eun Yu[1,2], Chan Park[1,2], Yeon-Sook Kim[4], Nam-Hyuk Cho[5], Jun Chang[3], Baik L. Seong[1,2]*

* Correspondence: Baik L. Seong (E-mail: blseong@yonsei.ac.kr)

Supplementary Figures and Tables

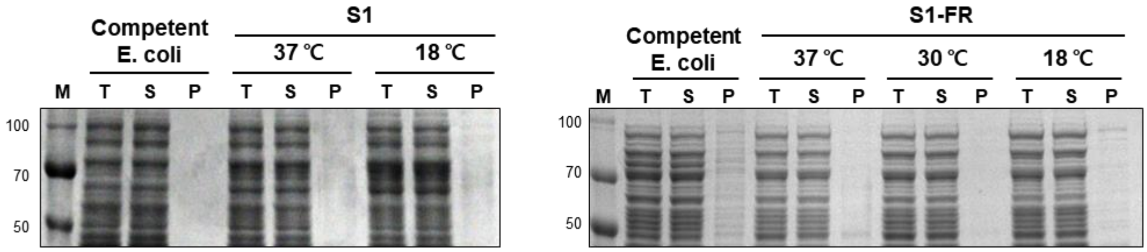

Supplementary Figure 1. The solubility of the S1 and S1-FR protein by SDS-PAGE analysis. The expression level of the protein was below the detection level.

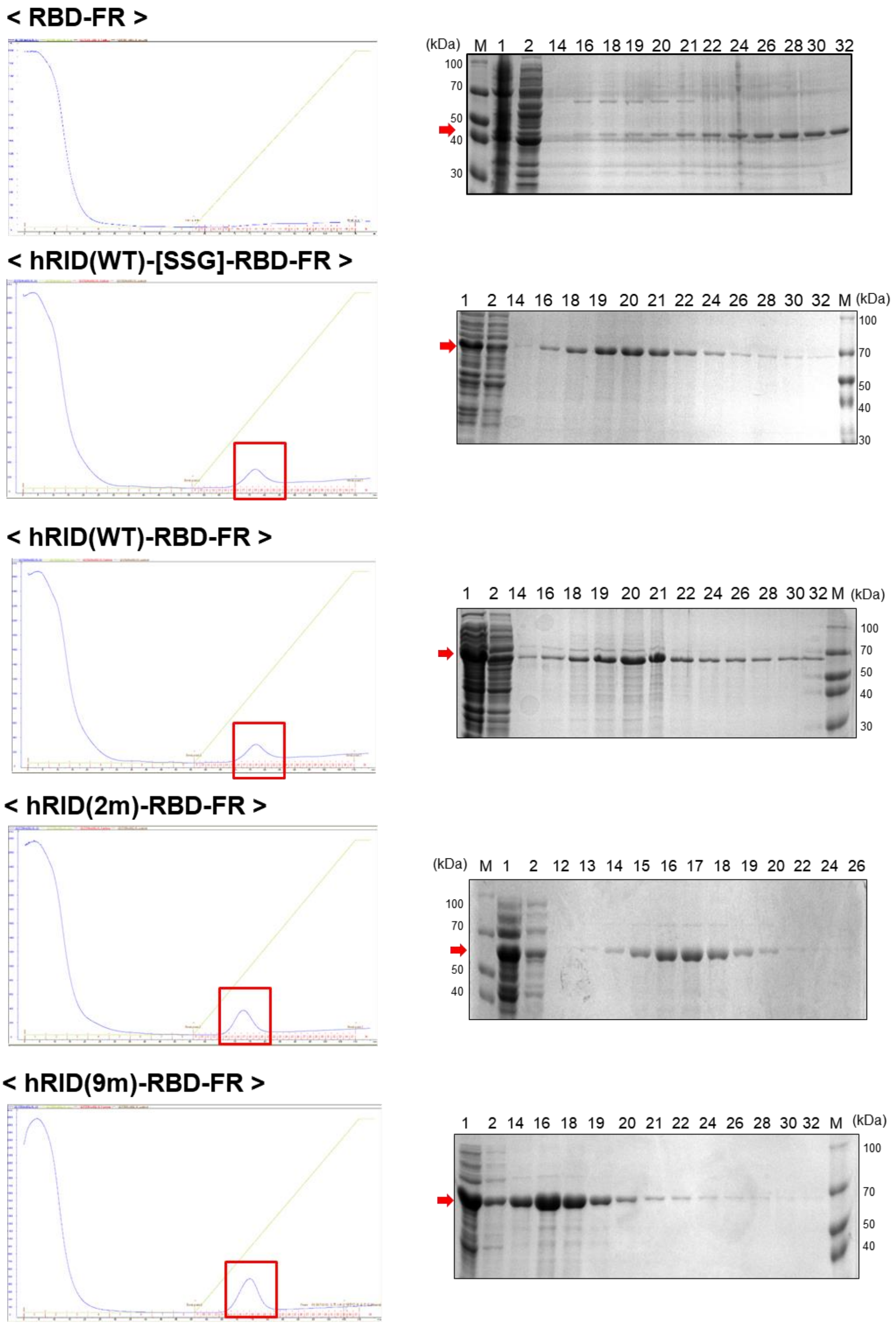

Supplementary Figure S2. Purification of RBD-FR, hRID(WT)-RBD-[SSG]-FR, hRID(WT)-RBD-FR, hRID(2m)-RBD-FR and hRID(9m)-RBD-FR using nickel affinity chromatography. Red arrows and red boxes indicate purified proteins.

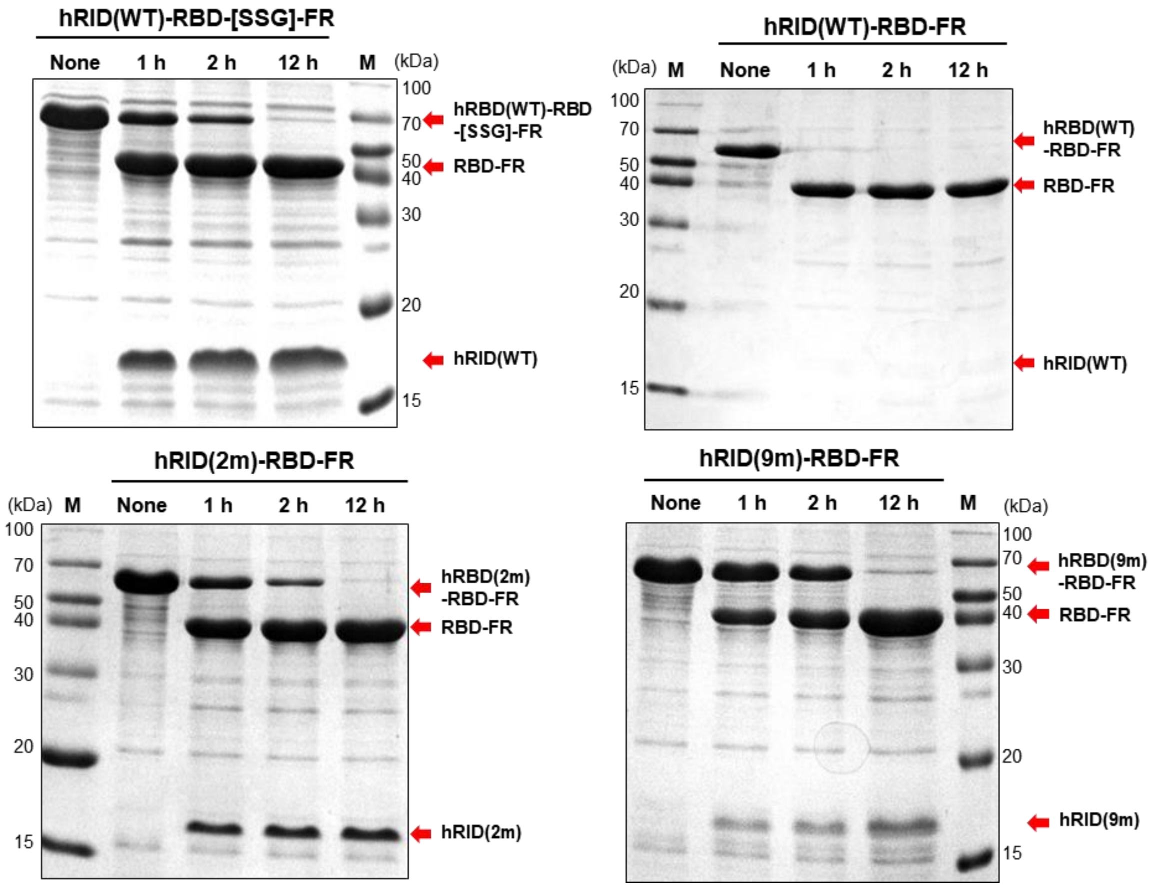

Supplementary Figure S3. TEV protease cleavage of hRID fused RBD-FR proteins. M, none, 1 h, 2 h, and 12 h represent molecular weight marker, no TEV treated, 1 hour, 2 hours, and 12 hours after TEV treatment, respectively. The whole protein, TEV-cleaved RBD-FR, and TEV-cleaved hRID were shown as red arrows.

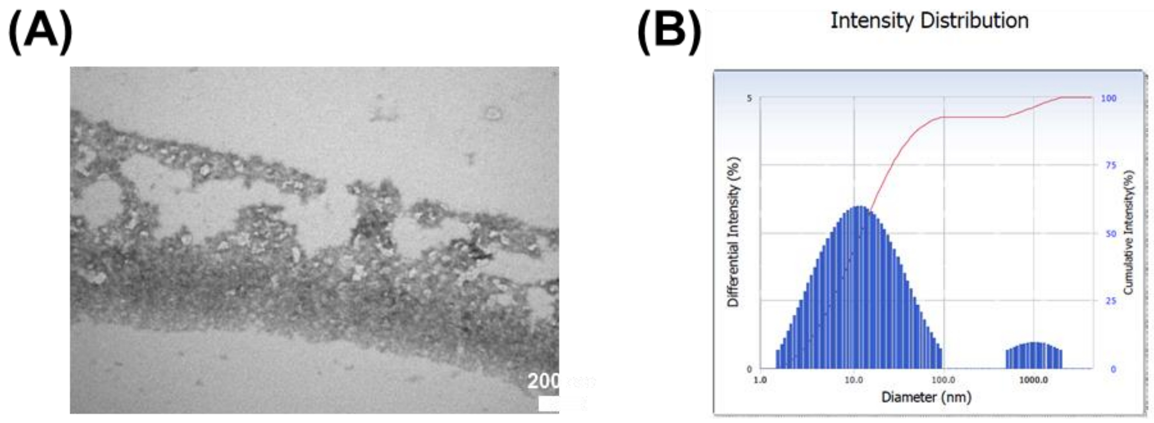

Supplementary Figure S4. The morphology of NPs of the purified hRID(WT)-RBD-FR was examined by TEM and DLS.

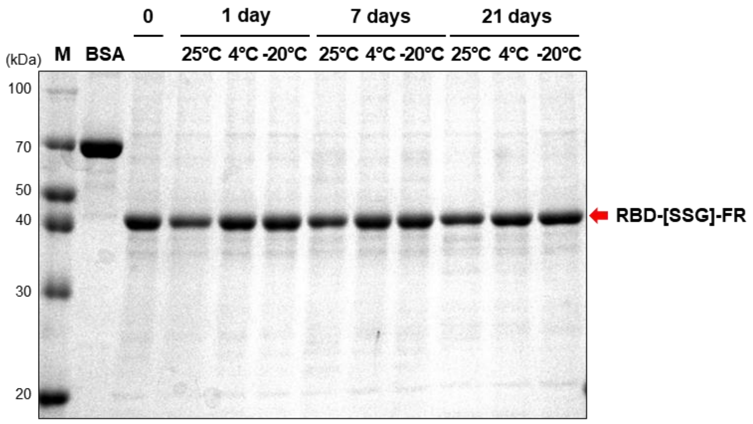

	0 DAY	1 DAY			7 DAYS			21 DAYS		
Temperature (°C)		25	4	-20	25	4	-20	25	4	-20
Protein (μg)	2.1	1.61	2.0	2.1	1.6	1.99	2.04	1.48	1.95	2.02

Supplementary Figure S5. The stability of purified RBD-[SSG]-FR under various period (0, 1, 7, and 21 days) and temperatures (25, 4, and -20 °C). The amount of proteins was calculated by comparing the band intensity of BSA (0.5 mg/ml) using the software 'Bio1D' (VILBER).

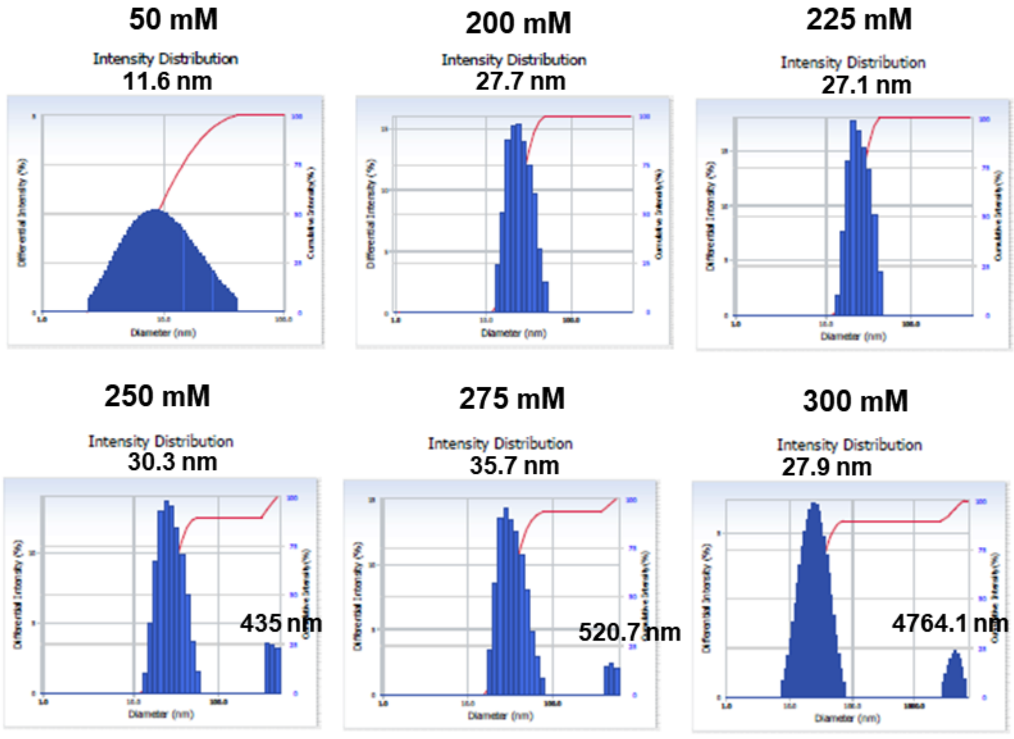

Supplementary Figure S6. DLS analysis of RBD-[SSG]-FR depending on NaCl concentrations (50, 200, 225, 250, 275, and 300 mM). The purified proteins were further used for confirming NPs formation by DLS.

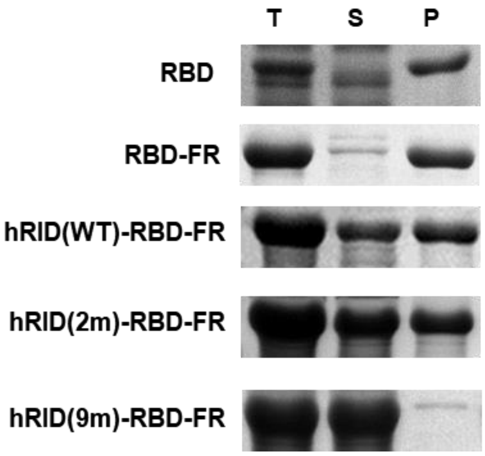

Supplementary Figure S7. SDS-PAGE analysis of 5 proteins (RBD, RBD-FR, hRID(WT)-RBD-FR, hRID(2m)-RBD-FR, and hRID(9m)-RBD-FR).

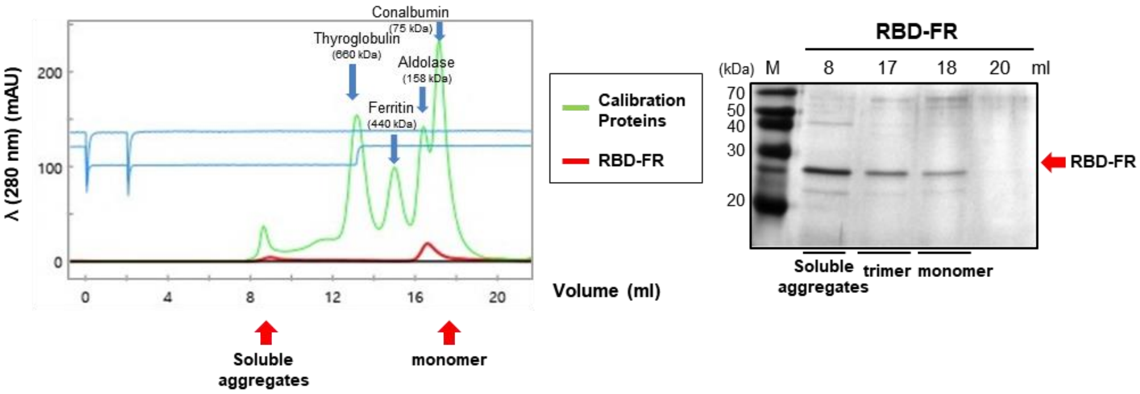

Supplementary Figure S8. SEC analysis of RBD-FR without hRID fusion. Fractions, each representing different multi-molecular assemblies of monomers were compared by SDS-PAGE.

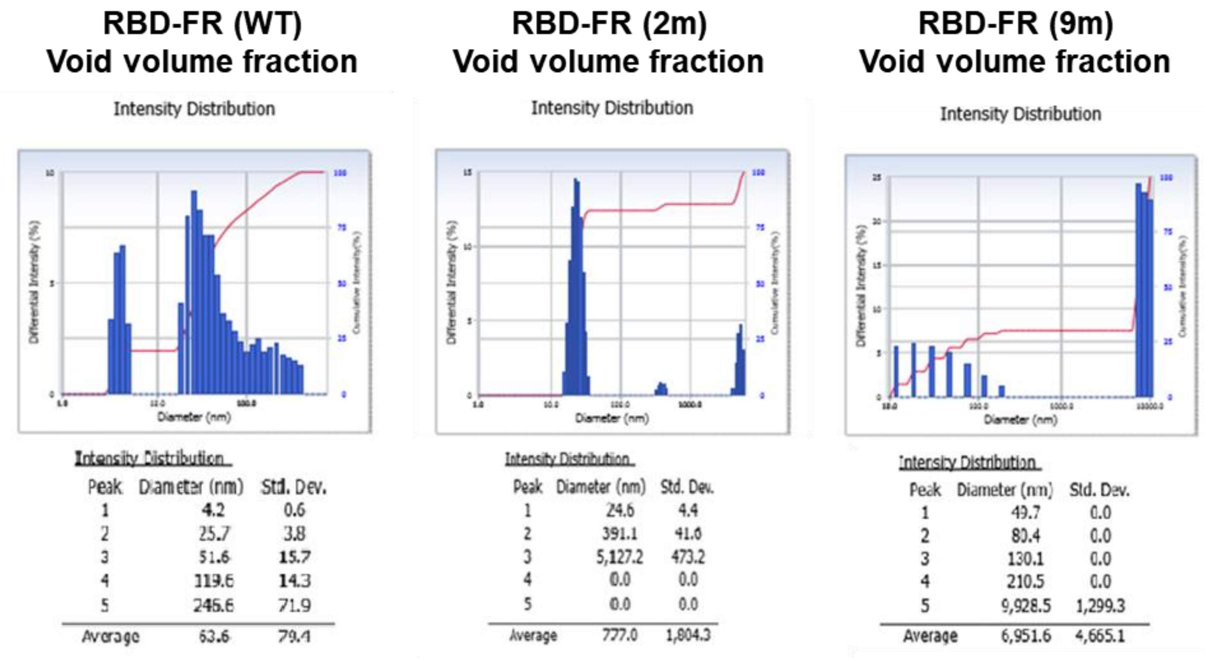

Supplementary Figure S9. Intensity distribution diameter of void volume of RBD-FR (WT, 2m, and 9m) as determined by DLS. The void volume of RBD-FR (WT, 2m, and 9m) from SEC (Figure 6A) were used for DLS analysis.

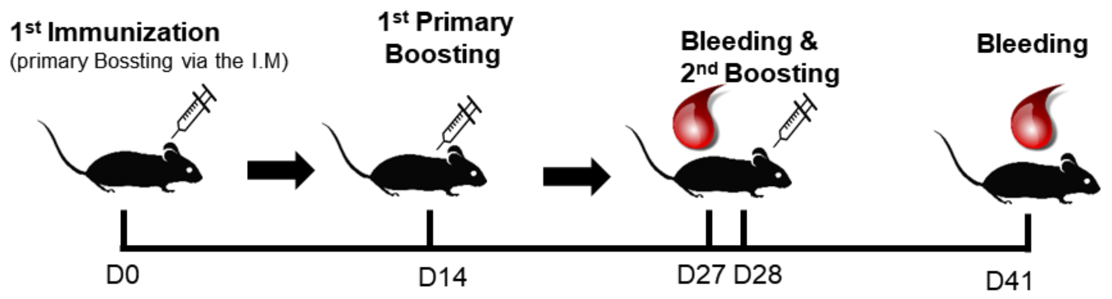

Supplementary Figure S10. Immunogenicity of the antigens with adjuvant (MF59 and alum). Schematic illustration of the immunization via I.M (intramuscular injections) and ocular bleeding from the orbital sinus schedule.

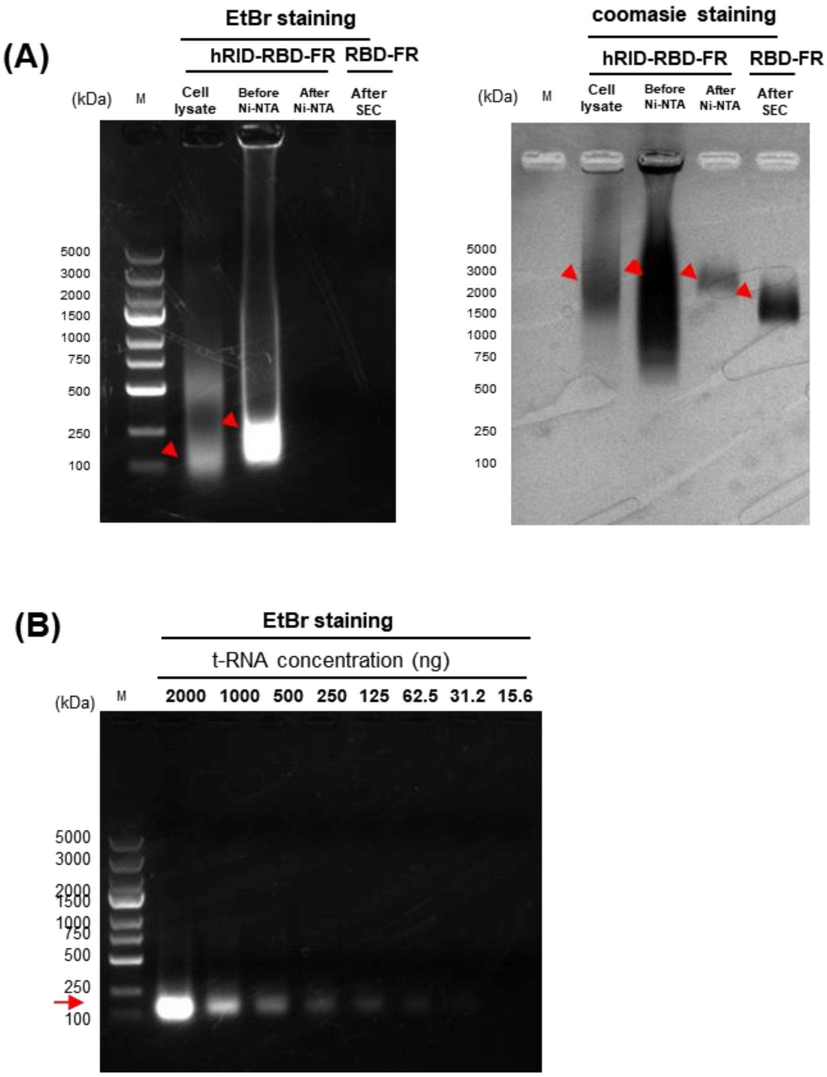

Supplementary Figure S11. Identification of RNA contamination of protein. (A) Identification of RNA contamination of cell lysate, hRID-RBD-FR and RBD-FR. The red arrow on the EtBr-stained gel and Coomassie blue-stained gel represent the co-purified RNAs and purified proteins, respectively. Antigens were found to be below detection level (31.2ng). All proteins were purified using Ni-NTA Resin and SEC. (B) Detection level of RNAs in the EtBr stained gel.

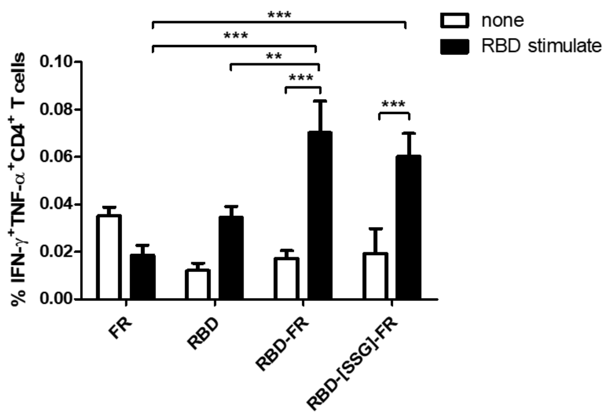

Supplementary Figure S12. Cellular immune response induced by RBD NPs-immunized mice. Specific IFN-γ and TNF-α responses in $CD4^+$ T cells of mice splenocytes were analyzed by flow cytometry. All data in graph were presented as means± s.d (n=4). P values were set using Two-way ANOVA Bonferroni posttests (**p< 0.01, ***p< 0.001).

**hRID(W) amino seq**	HMSEQHAQAAVQAAEVKVDGSEPKLS**K**NEL**KK**RL**K**AE**KK**VAE**K**EA**K**Q**K**ELSEKQLSQATAAATNHTTDNGVGPEEESVDDDDDDSGENLYFQGTGSDIVDKLHHHHHH*
**hRID(2m) amino seq** (K19A/K23A)	HMSEQHAQAAVQAAEVKVDGSEPKLSKNEL*A*KRL*A*AEKKVAEKEAKQKELSEKQLSQATAAATNHTTDNGVGPEEESVDDDDDDSGENLYFQGTGSDIVDKLHHHHHH*
**hRID(9m) amino seq** (K19A/K23A/R24A/K27A/K30A/K31A/K35A/K38A/K40A)	HMSEQHAQAAAVQAAEVKVDGSEPKLS*A*NEL*AA*RL*A*AE*AA*VAE*A*EA*A*Q*A*ELSEKQLSQATAAATNHTTDNGVGPEEESVDDDDDDSGENLYFQGTGSDIVDKLHHHHHH*

Supplementary Table1. The sequence of hRID point mutations affecting the RNA binding ability.
